# Evidence of evolutionary history and selective sweeps in the genome of Meishan pig reveals its genetic and phenotypic characterization

**DOI:** 10.1093/gigascience/giy058

**Published:** 2018-05-15

**Authors:** Pengju Zhao, Ying Yu, Wen Feng, Heng Du, Jian Yu, Huimin Kang, Xianrui Zheng, Zhiquan Wang, George E Liu, Catherine W Ernst, Xueqin Ran, Jiafu Wang, Jian-Feng Liu

**Affiliations:** 1 National Engineering Laboratory for Animal Breeding; Key Laboratory of Animal Genetics, Breeding, and Reproduction, Ministry of Agriculture; College of Animal Science and Technology, China Agricultural University, Beijing, 100193, China; 2 Department of Agricultural, Food & Nutritional Science, University of Alberta, Edmonton, T6G 2C8, Canada; 3 Animal Genomics and Improvement Laboratory, BARC, USDA-ARS, Beltsville, MD 20705-2350, USA; 4 Meat Animal Research Center, USDA-ARS, Clay Center, NE 68933 USA; 5 School of Animal Science, Guizhou University, Guiyang, 550025, China

**Keywords:** large-scale sequencing, Meishan pig, selective sweep, fecundity, *IGF1R*, *MCIR*

## Abstract

**Background:**

Meishan is a pig breed indigenous to China and famous for its high fecundity. The traits of Meishan are strongly associated with its distinct evolutionary history and domestication. However, the genomic evidence linking the domestication of Meishan pigs with its unique features is still poorly understood. The goal of this study is to investigate the genomic signatures and evolutionary evidence related to the phenotypic traits of Meishan via large-scale sequencing.

**Results:**

We found that the unique domestication of Meishan pigs occurred in the Taihu Basin area between the Majiabang and Liangzhu Cultures, during which 300 protein-coding genes have underwent positive selection. Notably, enrichment of the FoxO signaling pathway with significant enrichment signal and the harbored gene *IGF1R* were likely associated with the high fertility of Meishan pigs. Moreover, *NFKB1* exhibited strong selective sweep signals and positively participated in hyaluronan biosynthesis as the key gene of NF-kB signaling, which may have resulted in the wrinkled skin and face of Meishan pigs. Particularly, three population-specific synonymous single-nucleotide variants occurred in *PYROXD1*, *MC1R*, and *FAM83G* genes; the T305C substitution in the *MCIR* gene explained the black coat of the Meishan pigs well. In addition, the shared haplotypes between Meishan and Duroc breeds confirmed the previous Asian-derived introgression and demonstrated the specific contribution of Meishan pigs.

**Conclusions:**

These findings will help us explain the unique genetic and phenotypic characteristics of Meishan pigs and offer a plausible method for their utilization of Meishan pigs as valuable genetic resources in pig breeding and as an animal model for human wrinkled skin disease research.

## Background

The comparison of the Duroc pig (*Sus scrofa*) genome with whole-genome sequences from different pig populations provides a favorable opportunity to trace the history of pig domestication and to exploit evidence of long-term gene flow and artificial selection [1]. Genome sequencing indicates that a deep phylogenetic split between European and Asian wild boars happened approximately one million years ago [1]. Subsequently, approximately  10,000 years ago, pigs were domesticated at multiple locations across Eurasia [2]. With sequencing costs dropping, several recent studies have explored the origin, domestication, and evolutionary bottleneck of European and Asian native pigs [3–5]. Previous studies demonstrated that the distinct phenotypic characteristics between European and Asian pig breeds were due to the independent domestication of local wild boar populations in Asia and Europe. After the split between European and Asian pigs, the gene flow between Eurasian wild and domestic pig genomes, and human-mediated introgression have affected breed haplotypes [6, 7]. Especially, artificial selection affected behavior and morphology and led to different domestic traits in European and Asian pigs [6, 8, 9].

Among Asian pigs, the Meishan pig, named for the Chinese prefecture of Meishan, is well known as one of the most prolific breeds in the world. Besides their high fecundity, Meishan pigs have characteristics of early maturity, large drooping ears, and wrinkled black skin, which differ from those of other pig breeds. The unique features of the Meishan pigs have received wide attention, and several studies have focused on the identification of genetic diversity and population structure to unravel functional genes underlying their superior reproductive ability [10–12]. However, due to the high complexity of fecundity and related phenotypes, the genetic basis of these characteristics in Meishan pigs, particularly at the genomic level, remains largely unknown.

Following the idea that the development of the characteristics of a domesticated species is mainly caused by unique adaptive evolution to changing the climate and artificial selection [13], it can be presumed that the evolutionary history of Meishan owing to both natural and artificial selection could explain its specific biological characteristics. Therefore, to find potential genomic evidence linking the domestication of Meishan pigs with their breed characteristics, we performed a large-scale sequencing and systematic comparisons between 32 unrelated Meishan pigs and 86 other wild and domesticated pigs. We identified genomic evidence for the adaptive evolutionary history of the Meishan population and explored a suite of promising genes with Meishan-specific genomic variants and those having undergone positive selection in Meishan genome. The findings herein will provide insights to increase understanding of the genetic base that determines the unique traits of Meishan pigs. These findings should lay a solid foundation to utilize the valuable genetic resources of Meishan pigs for pig breeding and production, as well as to simulate future genetic studies.

## Results

### Genomic variant identification in Meishan pig breeds

**Figure 1:**
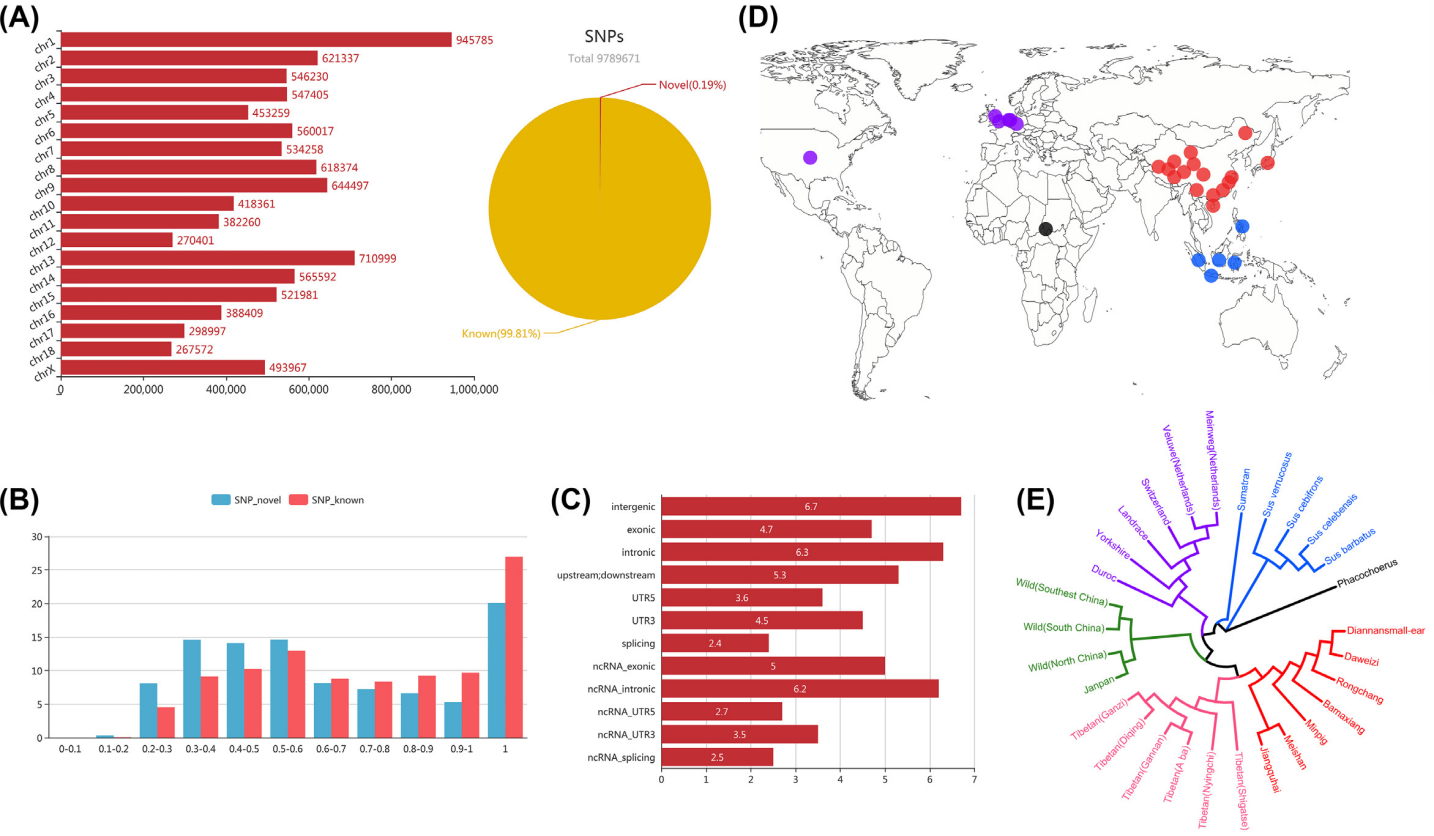
SNV characteristics of Meishan pig and the geographic and genetic relationship for 29 representative pig breeds. A) Nucleotide diversities of Meishan pig breeds and their presence within the dbSNP database. Bar plots represent the number of SNVs. Pie charts show the percent of Meishan SNVs within the dbSNP database. B) The relationship between known and novel variants with various allele frequencies. Bar plots represent the percentage (%) of genetic variations within various allele frequencies. C) Gene annotation of genetic variations. Bar plots represent the number of genetic variations (log_10_) within various functional regions. D) Geographic origin of 29 analyzed pig breeds. Twenty-nine analyzed subspecies from the four main geographic groups were collected from Europe and America (n = 6; purple circles), Africa (n = 1; black circle), Asia (n = 17; red circles), and the Southeast Asia (n = 5; blue circles). E) Neighbor-joining tree constructed from SNV data among 29 subspecies.

To detect genome-wide variation in Meishan pig breeds, we performed whole-genome resequencing of 32 unrelated Meishan pigs aligned against the *Sus scrofa* 10.2 reference genome using the Burrows-Wheeler Alignment tool (BWA) [14]; this generated a total of 732.76 Gb of sequence data with > 8× mapped read depth on average (Table S1). Whole-genome single-nucleotide variants (SNVs) were identified at the population level using the same variant detection pipelines and rigorous filtration criteria as used in our previous study [15]. A total of  9,789,671 SNVs with high quality were detected in the Meishan population (Fig. 1A), of which  18,366 SNVs were newly identified (not included in the dbSNP database [16]). These novel SNVs were expected to be present at lower frequencies or to be specific to the Meishan population, accounting for their lack of prior detection (Fig. 1B).

Further annotation of these identified SNVs in the Meishan population (Fig. 1C) revealed that they were most abundant in the intergenic regions (~ 55.6%), followed by introns, upstream and downstream regions, exons, untranslated regions (UTRs), and splicing site regions. Interestingly, we observed that more SNVs were located in the intronic regions of protein-coding genes (PCGs) than in those of the long noncoding RNAs (lncRNAs); however, more SNVs were detected in the exonic regions of lncRNAs than in those of the PCGs, suggesting that the selective pressure in the exonic regions of the PCGs was stronger than in the other functional regions. We also observed more genetic variations in the 3′-UTRs (0.313% in the PCGs and 0.035% in the lncRNAs) than in the 5′-UTRs (0.043% in the PCGs and 0.005% in the lncRNAs) in both PCGs and lncRNAs (Fig. 1C). This pattern is similar to that in the human genome [17]. With respect to exonic regions, we found a total of  51,985 potential functional genetic variations, including  34,170 synonymous SNVs,  17,625 non-synonymous SNVs, 155 stop-gain SNVs, and 35 stop-loss SNVs. These potential functional SNVs will provide valuable genetic resources for further exploration of the genetic structure and selective signatures in the Meishan population.

### Population diversity and demographic history

To infer the demographic history and time of divergence of the Meishan population, we downloaded the sequence data of another 28 representative non-Meishan pig individuals, comprising 9 domestic pigs, 13 wild boars, 5 other *Sus* species, and one outgroup (*Phacochoerus africanus*) from different geographical locations across three continents (Fig. 1D). A neighbor-joining tree of the pigs inferred from all SNVs was consistent with the results of previous studies and demonstrated strong clustering of pigs according to four major branches previously outlined on the basis of geographic and genetic classification [8, 18] (Fig. 1E); this might be because the pigs originated from very close geographical areas with domestication occurring under similar conditions.

**Figure 2:**
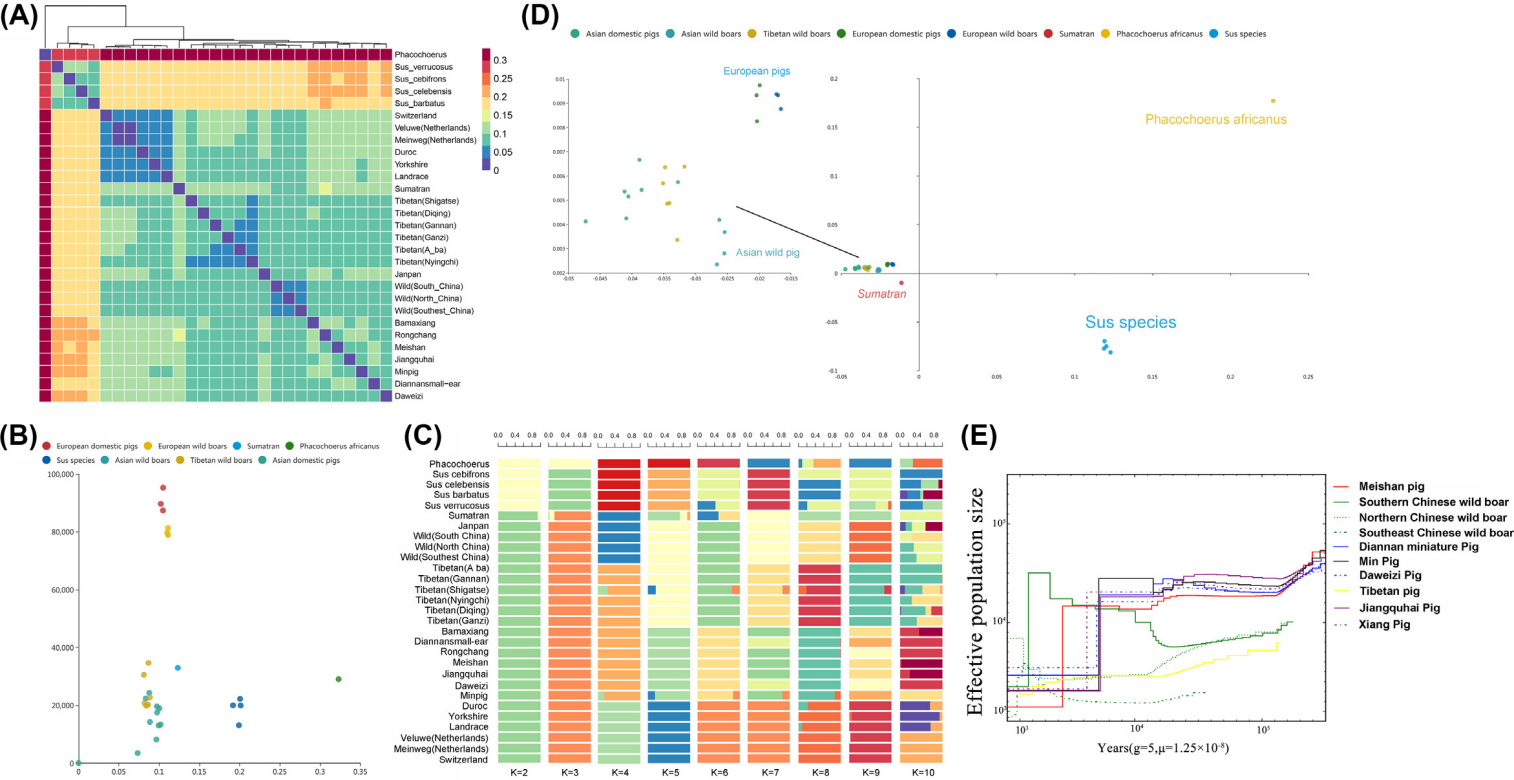
Population diversity and demographic history of Meishan pigs. A) The heatmap inferring the genetic relationship using SNV data from 29 subspecies. The heatmap was used to reveal the genetic distance between pairwise subspecies. B) Scatter diagram showing the relationship between geographic and genetic distance among 29 subspecies. The X-axis represents the genetic distance and Y-axis represents the geographic distance. C) ADMIXTURE analysis showing clustering of samples from 29 subspecies within K groups. K refers to the number of presumed ancestral groups. D) PCA plot with SNV data. Different colors represent different subspecies. E) Demographic history of Meishan and other Chinese domestic and wild pigs. Generation time (g) = 5 year and transversion mutation rate (u) = 1.25 × 10^−8^ mutations per bp per generation.

The distribution of genetic distance (Fig. 2A) indicated that pigs from the same habitat were more likely to have similar genetic distance and the clearest clusters. However, further comparison of the geographical distance and genetic distance of these non-Meishan breeds with the Meishan breed (Fig. 2B) revealed only weak correlation (*cor* = 0.41, *P* = 0.028). This suggests that the degree of genetic distance between different populations is determined not only by geographical isolation but also by the speciation time and human intervention. As shown in Fig. 2B, gene flow from Asian pig breeds to European breeds by means of artificial selection led to a relatively strong genetic relationship between Meishan and European pigs (geographical distance/genetic distance =  85,336), especially domesticated European breeds (geographical distance/genetic distance =  90,758).

Based on genetic co-ancestry analyses [19], we partitioned all individuals into known groups by varying the number of presumed ancestral populations (Fig. 2C, K ranged from 2 to 10). When K was set to 4, four leading clusters were clearly observed: *P. africanus and* other *Sus* species; Western domestic and wild pigs; Asian domestic and Tibetan wild boars; and Asian and Sumatran wild pigs. When K was set to 5, Tibetan wild boars could be separated from Asian domestic pigs. Interestingly, we also found that *Sus verrucosus* had more genetic exchange with *Sus scrofa* (Sumatran) than other *Sus* species, likely owing to recent human-mediated activities [20]. For values of K < 10, Meishan pigs were distinguishable from Asian domestic breeds and shared some genetic information with Jiangquhai pigs. Ternary principal component analysis (PCA) plots were also constructed with SNVs, revealing very similar patterns (Fig. 2D) to those identified by ADMIXTURE software [21] with K = 3.

As the unique genetic characteristics of Meishan pigs might be related to distinct divergence events, we further conducted a multiple sequentially Markovian coalescent (MSMC) analysis [22] for Meishan pigs and six other Chinese domestic populations as well as three Chinese wild boar populations, to infer historical changes in effective population size (*Ne*). A declining tendency in population size was detected in seven Chinese domestic pig populations through 7.2–4 kyBP (kilo years before present; Fig. 2E); this period largely encompassed the post-glacial stage when temperatures appeared to be increasing and humans were moving into the modern period. In fact, the warm climate was beneficial to both development of human civilization and the domestication of pigs, showing that human-driven artificial selection may result in a “bottleneck” in the evolution of different domesticated breeds. Most interestingly, unlike the other six Chinese domestic pig populations, Meishan pigs showed a later bottleneck, with the occurrence of a marked bottleneck 4,000–5,000 years ago (red line, Fig. 2E), reflecting when Meishan breeds likely underwent a unique domestication process. More precisely, in the Taihu Basin area, three cultures were recorded during this period (7–4 kyBP): the Majiabang Culture (7–6 kyBP), Songze Culture (6–5 kyBP), and Liangzhu Culture (5–4 kyBP). Archaeological evidence indicates the presence of pigs at the site of the Taihu Basin approximately 7,040 years ago [23]. We accordingly inferred that the unique domestication of Meishan pigs in the Taihu Basin area started from the Majiabang Culture and continuously developed as late as the Liangzhu Culture.

### Population structure and selection sweeps

**Figure 3:**
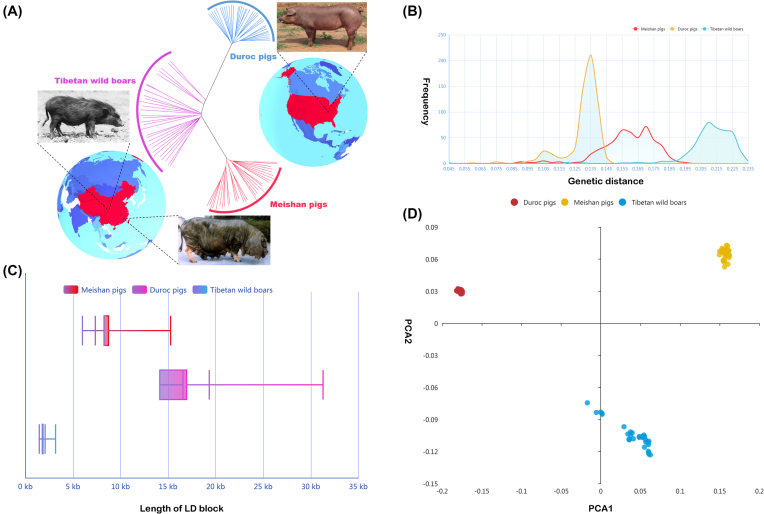
Genetic relationships and population structure among three pig populations. A) Neighbor-joining phylogenetic tree of three pig populations. The genetic distance is measured by SNV data from Meishan, Duroc, and Tibetan wild boar populations. B) Frequency distribution of genetic distance among three pig populations. The X-axis represents the genetic distance and Y-axis represents frequency. C) Length distribution of LD block among three pig populations. Red, purple, and blue bars represent Meishan, Duroc, and Tibetan wild boar populations, respectively. D) PCA plot for three pig populations with SNV data. Different colors represent different pig populations.

To mine in depth the genomic evidence contributing to the breed features of Meishan pigs, we further compared the genomic signatures of Meishan pigs at the population level with those of two other typical pig breeds with characteristics greatly differing from Meishan pigs (*i.e*., 30 Tibetan wild boars as representatives of Asian wild boar populations, and 35 Duroc pigs representing European domesticated breeds). Genetic distinctiveness among these pig populations reflected the pattern of isolation by their adaptation/environment (Fig. 3A). The intrapopulation genetic distance for each breed was considerably smaller than the inter-population genetic distance between the different breeds (Fig. 3B). Furthermore, the intra-population genetic distance of the domesticated pig breeds was lower than that of the Tibetan wild boars. This demonstrates that artificial selection tends to reduce genetic diversity, and commercial breeds have undergone stronger artificial selection than have local breeds. The impact of artificial selection is also reflected in the genome linkage disequilibrium (LD) levels in each population (Fig. 3C), indicating that artificial selection can facilitate the increase of LD within a population [24]. PCA also revealed a similar pattern (Fig. 3D), where Meishan population was shaped in a tight cluster and was clearly separated from the other populations.

**Figure 4:**
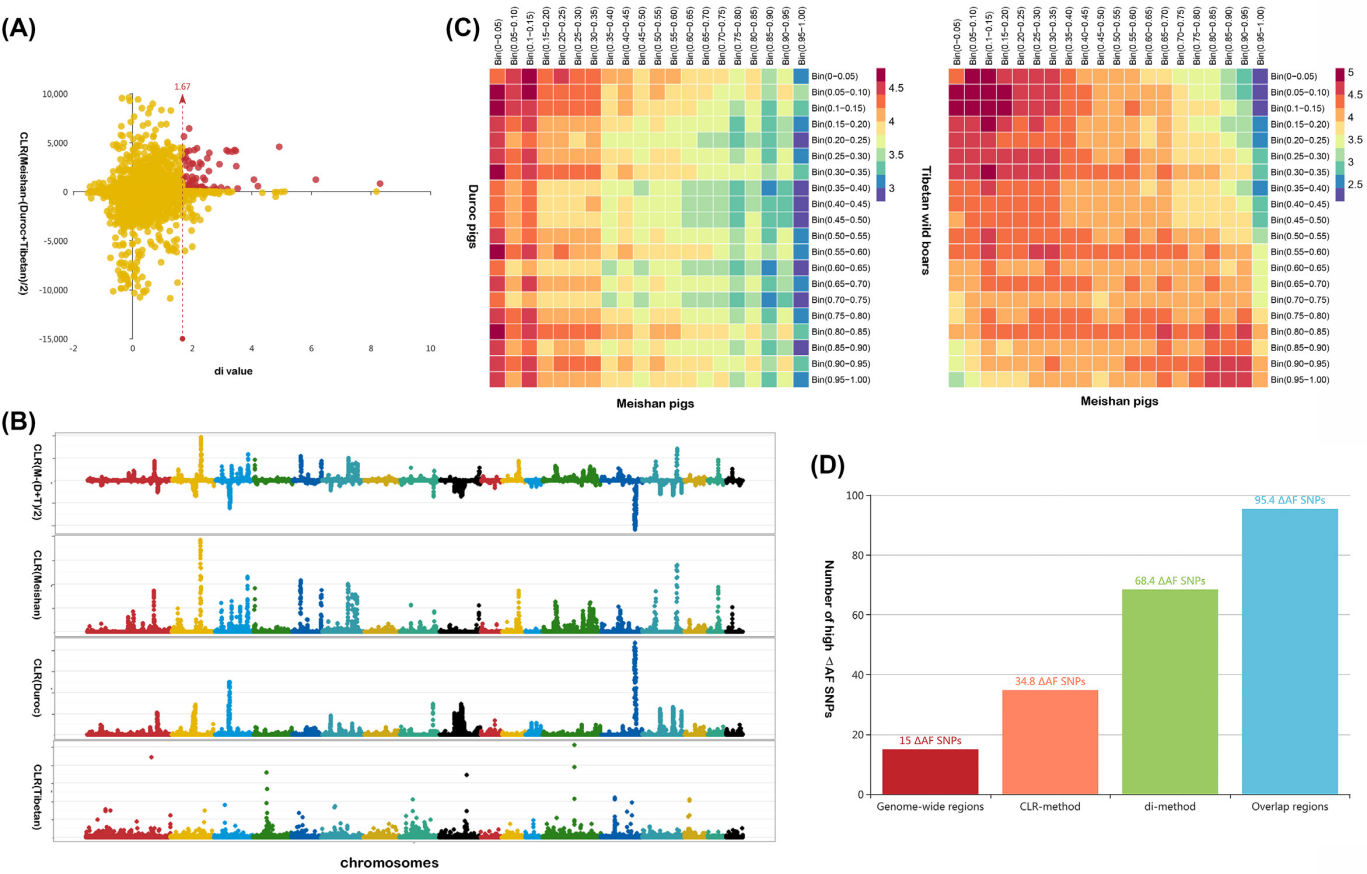
Selection sweeps of Meishan population. A) Definition of sweep regions for Meishan population. The X-axis represents the D_i_ value and Y-axis represents CLR value. Red circles mean sweep regions for Meishan population. B) The plot of CLR values among three pig populations. Part 1 shows the genome-wide distribution of ΔCLR; Part 2–3 represent the genome-wide distribution of CLR signal values for Meishan, Duroc, and Tibetan wild boar, respectively. C) Quantitative distribution of SNVs between Meishan and other population within various bins. The heatmap showing the number of SNVs (log_10_) with a bin in steps of 0.05. D) A number of high ΔAF SNVs within various specific regions. The overlap regions represent the overlap between di-method and CLR-method based regions.

Based on the population-scale genetic differences between Meishan and the other pig breeds, we speculated that there should be specific genome signals in the Meishan population arising from long-term artificial and positive natural selection during domestication. To further identify the genomic locations of these selective sweeps in the Meishan pigs, we calculated the genome-wide statistic *d_i_* [25] for the Meishan to Duroc and Tibetan wild boar populations. Focusing on the regions at the top 1% of the *d_i_* empirical distribution (Fig. 4A), we identified 197 significant regions (*d_i_* > 1.672) harboring 300 candidate PCGs (204 functional annotated genes) and 171 lncRNA genes (Table S2–3). Among these genes, composite likelihood ratio (CLR) tests [26] revealed that a major proportion of PCGs (57.33%) and lncRNAs (53.22%) also fell into regions of selective sweeps with stronger positive selection signals in Meishan pigs than in other breeds (Fig. 4B); these regions commonly identified by both CLR and *d_i_* statistic may be potentially related to selection during the domestication of Meishan pigs.

As the highly differentiated SNVs across populations more readily occurred in the vicinity of the region under selection [27], we further compared the alternate allele frequency (the introduced new allele) of all identified SNVs in the Meishan population with those in the other two populations. Quantitative distributions of SNVs in the Meishan and the other two populations was surveyed by grouping all SNVs with frequencies harbored in the corresponding interval in steps of 0.05 (i.e., 0–0.05, 0.05–0.10, etc. until 0.95–1.00) (Fig. 4C). We accordingly calculated the absolute allele frequency difference [ΔAF = abs {AltAF_Meishan_ − mean (AltAF_Duroc_ + AltAF_Tibetan_)}] between the Meishan population and the other two populations to assess the potential selective sweeps of Meishan breeds. We determined that  56,305 breed-specific SNVs were merely fixed in the Meishan population (ΔAF = 1). Significant enrichment was noted for high-ΔAF SNVs (>0.8) within the identified sweep regions, particularly the overlapping regions identified using both CLR and *d_i_* methods (Fig. 4D); this reflected the fact that the highly differentiated population SNVs were actually associated with artificial and natural selection. Further comparisons of ΔAFs per 0.05 bins within various functional regions (exonic, intronic, UTRs, *etc*.) (Table S4–5) revealed significant enrichment for low-ΔAF SNVs (0.1 <ΔAF<0.35, *χ^2^* test, *P* < 9.98 × 10^−7^) in exonic regions, but significant enrichment for high-ΔAF SNVs (0.55 <ΔAF<1, *χ^2^* test, *P* < 0.0014) in intronic regions. Notably, for the SNVs in exonic regions, we observed a significant excess of synonymous SNVs within different ΔAF bins (0.3 <ΔAF < 0.8, *χ^2^* test, *P* < 0.0012), but non-synonymous SNVs were largely enriched in the low-ΔAF bin (0 <ΔAF < 0.15, *χ^2^* test, *P* < 0.0012). The results support the supposition that most of the genetic changes during domestication are concentrated in the regulatory regions rather than the coding regions [27].

### Meishan-derived introgression in European pigs

Meishan pigs, as the major Chinese pig breed, contributed considerably to improving commercial production in European pig breeds during the Industrial Revolution [7]. Here we identified the present region of introgressed Meishan haplotypes in European domestic pigs (Duroc) using the pairwise identical by descent (IBD) method [7]. We calculated the normalized IBD (nIBD) for each  10,000-bp bin in the pig genome to estimate the extent of introgression events, and the top 5% of nIBD regions were regarded as evidence of Meishan-derived introgression into European pigs.

**Figure 5:**
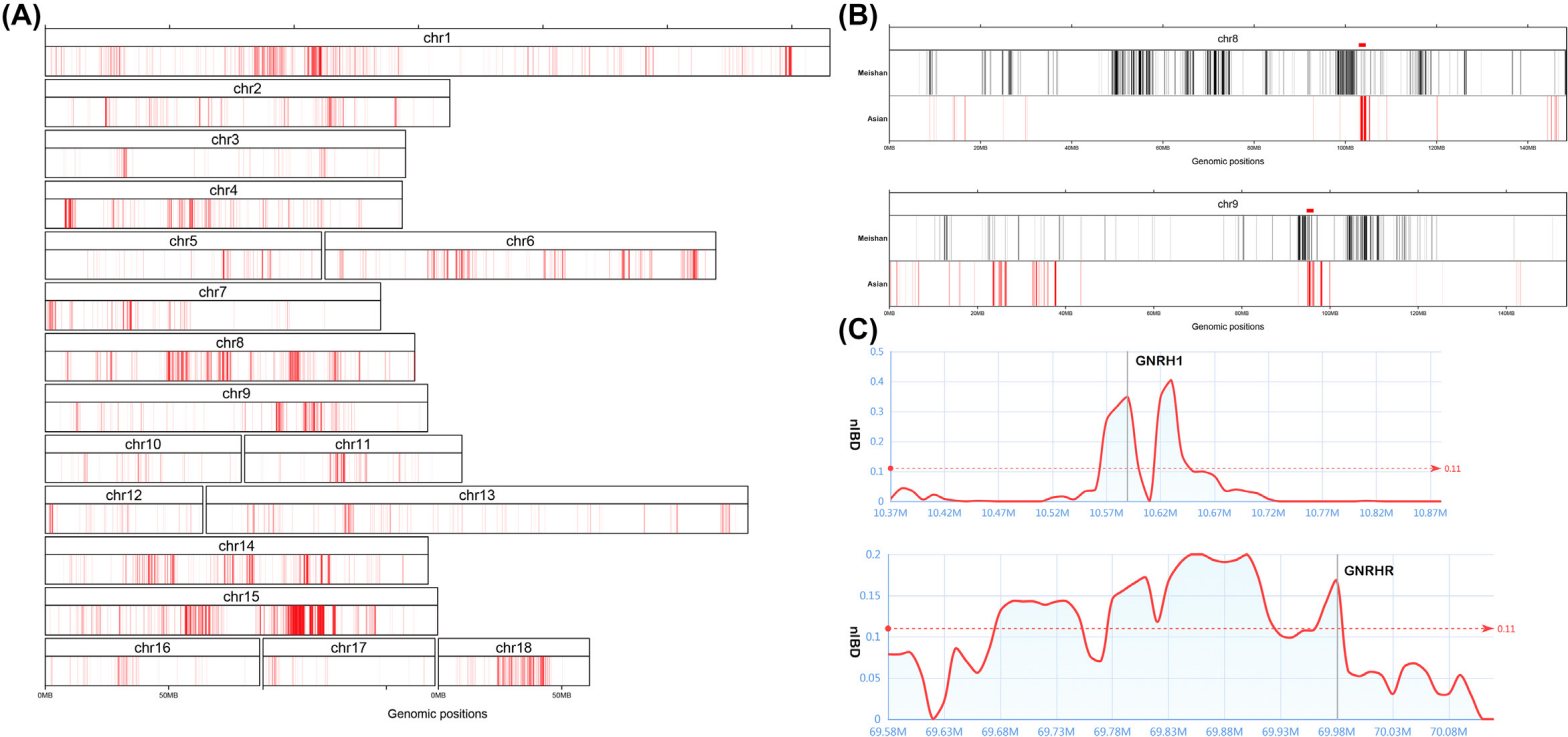
Meishan-derived introgression in European pigs. A) The map of Meishan-derived introgression in European pigs. The red bars represent the Meishan-derived introgression regions within chromosome 1–18. B) Two confirmed longest Asian-derived regions. The black bars represent the Meishan-derived introgression, and the red bars represent the Asian-derived introgression. C) *GNRH1* and *GNRHR* genes in Meishan-derived introgression region. Line charts represent the nIBD value distribution within Meishan-derived introgression region. The gray bar indicates the position of *GNRH1* and *GNRHR* genes.

We observed a total of  12,272 bins (122.7 Mb) (Fig. 5A) with an average nIBD value >0.10625 and finally merged 2,999 Meishan-introgressed regions with the length ranges from 10 to 1,430 kb (Table S6). Interestingly, of these, 3.44 Mb Meishan-derived regions (n = 121) were identified as Asian-derived introgression in European pigs by Groenen et al. [7] (Table S7). Remarkably, the presence of the two longest consecutive regions (in chromosomes 8 and 9) of Asian-derived introgression was confirmed in Meishan haplotypes (Fig. 5B). We also found introgression signals (nIBD > 0.2) of Meishan pigs on chromosome 9 near (∼190 kb) the aryl hydrocarbon receptor (*AHR*) gene, which was shown to be associated with female fertility and increased litter size in previous studies [7, 28]. We also observed some meat quality-related genes such as spalt like transcription factor 1 (*SAL1*) and malic enzyme 1 (*ME1*), which also shared more haplotypes with Asian domesticated pigs than with European wild boars. These findings demonstrate the contribution of Meishan pigs to the formation of Asian haplotypes in European pigs during the Industrial Revolution.

In addition to these shared haplotypes originating from Asian pigs, some regions of Meishan-derived haplotypes also provided potential evidence of Meishan fertile introgression. For instance, these regions contain the gonadotropin releasing hormone receptor (*GNRHR*) and gonadotropin releasing hormone 1 (*GNRH1*) genes, both of which are associated with hypogonadotropic hypogonadism and play an important role in reproduction (Fig. 5C). Particularly in the *GNRHR* gene, some SNVs have been associated with litter size in goats [29]. Therefore, the results further support that Meishan pig introgressions play a vital role in European haplotypes, especially sow fertility, and that these novel Meishan-derived introgressions could provide new insight into the artificial selection of modern European pig breeds.

### Characterization of candidate genes underlying breed features of Meishan pigs

**Figure 6:**
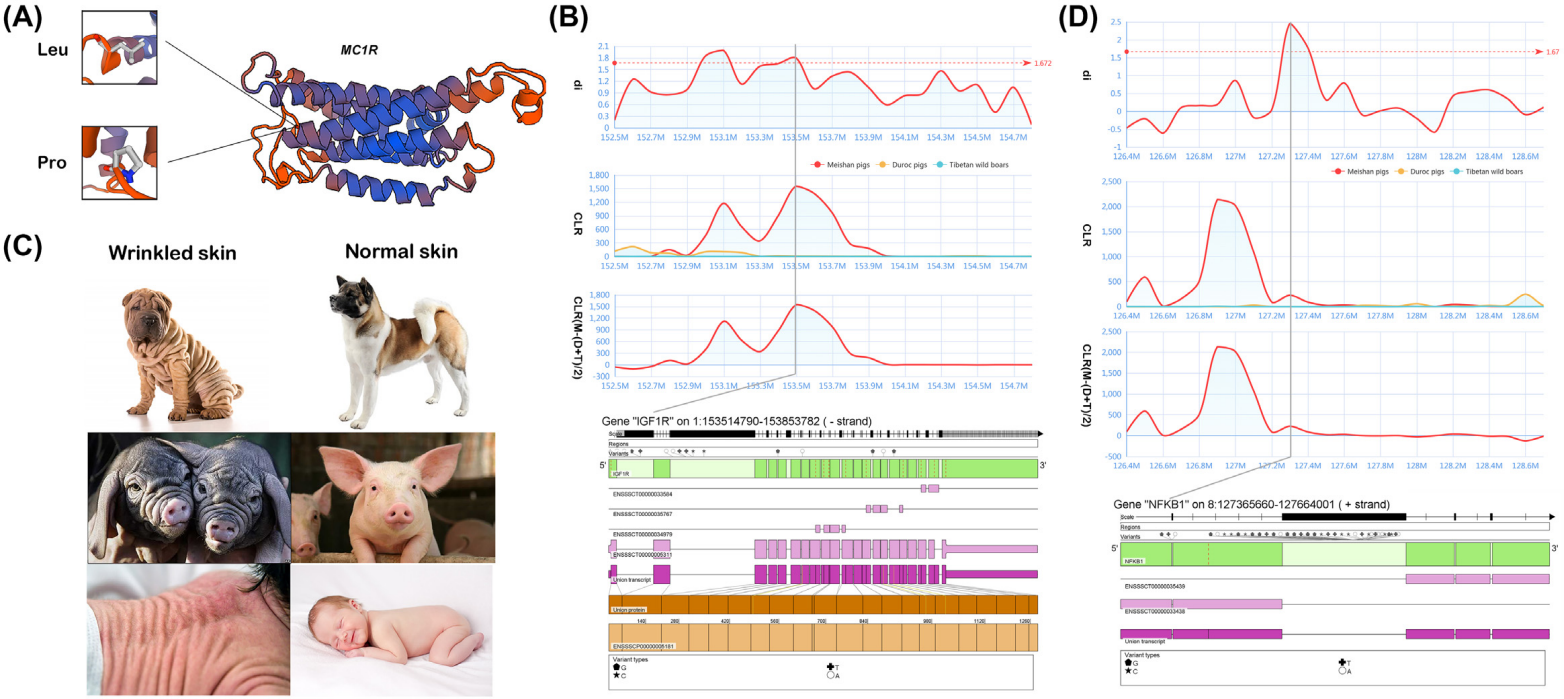
Specific genes with strong selective sweep signals in Meishan pigs. A) Prediction of protein conformation space for *MC1R*. Structure of Amino acids coded by exon 8 of porcine *MC1R*, as predicted by SWISS-MODEL. B) Candidate gene *IGF1R* for prolificacy in Meishan. D_i_ values, CLR values, and ΔCLR values are plotted surrounding *IGF1R* gene. The bottom part showing the gene structure and all high ΔAF SNVs within a gene. The gray bar indicates the position of the *IGF1R* gene. C) Comparison of phenotype between the wrinkled and unwrinkled skin. D) Candidate gene *NFKB1* for wrinkled skin and face of Meishan pig and the labelling is same to Fig. 6B.

Through comparison of gene frequencies between Meishan and the other two representative breeds, we identified a total of 280 candidate Meishan-specific SNVs in exonic regions with the criteria of AF_Meishan_ > 95% and AF_non-Meishan_ < 5% (Table S8). These SNVs mapped to the regions of 244 PCGs (132 functionally annotated genes). Of note, 114 of the 244 PCGs (52 functionally annotated genes) appeared at a higher evolutionary rate in Meishan breeds, and their coding structures were changed by 125 large-effect mutations: 123 nonsynonymous and 2 stop-gain SNVs. In particular, of these 125 large-effect mutations, 3 perfectly fixed synonymous SNVs (ΔAF = 1) occurred respectively in *PYROXD1*, *MC1R*, and *FAM83G* genes. Intriguingly, these three genes had been shown in studies on humans to play an important roles in the development of skeletal muscle [30], skin [31, 32], and bone [33]. Particularly, we found a missense SNV (exon1: c.T305C: p.L102P; rs45434630 in the dbSNP database) within the Melanocortin 1 receptor (*MC1R*) gene that generated a Leu-to-Pro substitution, leading to a change in the *MC1R* protein conformation space (Fig. 6A). The *MC1R* gene is mainly expressed in the melanocytes of hair follicles and controls melanogenesis, which has been shown to be associated with black (dominant E^D^) coat color pattern in pigs [31]. Therefore, the identification of this nonsynonymous mutation in *MC1R* helps us to better explain the black coat of the Meishan breed.

Aside from the aforementioned potentially fixed genes, we further collected all 300 PCGs (204 functionally annotated genes) with significant positive selection signals identified in the Meishan genome for follow-up pathway analyses. Kyoto Encyclopedia of Genes and Genomes (KEGG) enrichment analyses detected a total of 34 pathways harboring 120 of these 204 annotated PCGs (*P*< 0.05; Table S9). Intriguingly, the identified FoxO signaling pathway exhibited the best statistics for enrichment signals (corrected *P* = 0.022), with five positive selection-related genes involved (*ATM*, *CSNK1E*, *CCNB1*, *GABARAP*, and *IGF1R*). The FoxO signaling pathway regulates ovarian prostaglandins, which are critical for reproduction [34]. We further focused on the most promising gene, insulin-like growth factor 1 receptor (*IGF1R*), involved in the FoxO signaling pathway. Previous studies have reported *IGF1R* to be crucial for female fertility as it participates in steroidogenesis, follicle survival, and fertility in female mice [35, 36]. Although there has been no direct evidence that polymorphisms for *IGF1R* are associated with litter size, numerous *IGF1R* mutations have been shown to affect late prenatal and early postnatal growth restriction, perinatal growth velocity, and diminutive body size [37, 38]. A proper litter size of piglets ensures a higher number of surviving offspring [39]; this is reflected in the large litter sizes of Meishan pigs. As expected, strong selective sweep signals (*d_i_* value = 1.80; CLR > 965) and 17 high-ΔAF SNVs (ΔAF > 0.8; including 3 synonymous SNVs and 14 intronic SNVs) were noted in the *IGF1R* gene region of the Meishan genome (Fig. 6B). We conclude, therefore, that the *IGF1R* gene and corresponding FoxO signaling pathway may be promising candidates for prolificacy-related positive selection in the domestication of the Meishan population.

Another typical phenotypic characteristic of the Meishan breed is its wrinkled face and skin, which is also observed in Shar-Pei dogs and human patients with folding and thickening of the skin [40] (Fig. 6C). This abnormality of cutaneous tissue is mainly due to anomalies in hyaluronan (HA) metabolism; the high activity of HA synthase increases the activity of dermal fibroblasts and gradually leads to the formation of wrinkled skin [41]. Previous studies have used Shar-Pei dogs as research animal models to successfully identify the candidate gene (hyaluronan synthase 2; *HAS2*) responsible for the skin wrinkles in Shar-Peis [42], but no direct evidence exists currently for the role of this gene in the extreme thickening of the human skin so far. In contrast to the results of the Shar-Pei study, we did not detect *HAS2* (LOC100152156) in the selection region of the Meishan pigs. However, we observed strong selective sweep signals (*di* value = 1.77; CLR > 89) and 43 high-ΔAF SNVs (ΔAF > 0.8; including 1 synonymous SNV, 1 UTR3, 1 downstream, and 40 intronic SNVs) in the Nuclear Factor Kappa B Subunit 1 (*NFKB1*) gene region (Fig. 6D), which, as a the key gene involved in NF-κB signaling pathway, has been shown to regulate HA biosynthesis positively [43, 44]. These results may better explain why wrinkled skin largely occurs on the face and neck in Meishan pigs. Together with the finding in Shar-Pei dogs, we speculated that both *HAS2* and *NFKB1* can act as candidate genes likely associated with the incidence of wrinkled skin in humans, since these two genes were reportedly paly key roles in HA biosynthesis.

## Discussion

This study provides the first comprehensive large-scale re-sequencing and survey for the Meishan, a pig breed with the highest known prolificacy in the world. Our results, as well as the downloaded re-sequencing data, will support future in-depth analyses on population genetics, demographic history, genomic selection, introgression, and breed-specific genetic variations in pigs. The identification of selective sweep regions, introgression regions, and breed-specific genetic variations associated with superior fecundity in Meishan pigs may contribute toward molecular marker-based breeding for improved pig reproduction.

The present study revealed that Meishan pigs share a similar, but not identical, genetic background with other Asian pigs; this finding is consistent with the fact that there was a domestication bottleneck caused by human-driven artificial selection and distinct domestication approximately 4,000 ∼ 5,000 years ago. This period in the Taihu Basin overlapped with the Liangzhu Culture, when the climate was warm and dry, rice agriculture was developing, and the human population was rapidly increasing. The unique climate of the Liangzhu civilization and land conditions of the Taihu Basin had a considerable lasting effect on the domestication of the Meishan breed and prompted the improvement of prolificacy traits.

We identified 244 Meishan-specific fixed genes and 300 PCGs undergoing positive selection. Of note, enrichment of the FoxO signaling pathway and its related gene, *IGF1R*, may be the most promising genomic evidence explaining the high fertility of Meishan pigs. We found that *NFKB1* exhibited strong selective sweep signals and, as a key gene in the of NF-κB signaling pathway, it plays an important role in HA biosynthesis, which likely induces the wrinkled skin and face of Meishan pigs. These findings help explain the unique phenotypic characteristics of the Meishan pig and provide new insights into the causes of infertility and extreme thickening and folding of skin in humans.

We provide new evidence that Meishan pigs greatly contributed to the improvement in commercial traits in European pig breeds during the Industrial Revolution. Notably, 3.44 M regions were also supported by Asian-derived introgression, including the two longest consecutive regions (chromosome 8 and 9) and the *AHR* haplotype. Moreover, we identified some novel Meishan-derived introgression regions and genes (*e.g., GNRH1*, *GNRHR*); this information should provide insights into the artificial selection of modern European pig breeds. Meishan pigs are one of the main Asian domesticated pigs that were introduced into Europe. However, they still retained several of their breed-specific genetic variations. Approximately 58 known PCGs were found to be influenced greatly by breed-specific genetic variations in our study. For instance, the black coat in Meishan pigs can be explained by the presence of the T305C missense SNV in the *MCIR*. Therefore, these findings will be valuable for future studies on the Meishan breed.

Our findings will facilitate explanations of the unique characteristics of Meishan pigs and offer a plausible method for their utilization as valuable genetic resources in pig breeding. Obviously, these fertility-related markers could be used during selection to increase fertility in pigs and increase the number of live-born piglets. The potential role of *NFKB1* as a new biomarker helps us improve our understanding of human patients with folding and thickening of the skin. It is worth noting that three Meishan-specific synonymous SNVs, which were detected in *PYROXD1*, *MC1R*, and *FAM83G*, will provide future research directions to study Meishan-specific phenotypic traits. As mentioned above, *MC1R* has been shown to possess a key amino acid mutation that leads to the black coat of the Meishan breed. Although there have been few studies of *PYROXD1* and *FAM83G* in pigs, they have been associated with the development of skeletal muscle and bone in humans. Therefore, it is expected that *PYROXD1* and *FAM83G* will provide new insights into the slow growth traits of Meishan pigs in future studies.

### Conclusions

In summary, the increased knowledge of Meishan phenotype-related genes helps to improve our understanding of the underlying biological mechanisms contributing to fertility, black coat, wrinkled skin, and growth traits in pigs as well as in other mammals, including humans.

## Methods and Materials

### Sample collection and sequencing

We sequenced a total of 63 samples (32 Meishan pigs from Kunshan City of Jiangsu Province and 31 Durocs from Yancheng City of Jiangsu Province) in this study (Table S1). We used the Qiagen DNeasy Tissue kit (Qiagen, Germany) to extract genomic DNA from pig ear tissue, and verified the integrity and purity of DNA by agarose gel electrophoresis and A260/280 ratio. All suitable genomic DNA was sequenced using an Illumina HiSeq 2000 sequencing system at Novogene (Beijing, China). The Illumina DNA libraries (Paired-end, 2 × 125 bp) were constructed for 63 pig samples and 1403.35 G bases were generated.

In summary, a total of 118 pigs were selected from 10 domesticated breeds, 13 wild boars, and 5 other Sus species, as well as a genus of wild pig from a different geographical location. These included 32 Meishan pigs, 35 Durocs, 30 Tibetan wild boars, 3 European wild boars (Meinweg and Veluwe from the Netherlands and another from Switzerland), 3 Asian wild boars (from South, North, and Southeast China), and one each of the following: Yorkshire, Landrace, Japanese wild boar, Mini pig, Jangquhai, Bamaxiang, Rongchang, Diannan small-ear pig, Daweizi pig, Sumatran, *Susbarbatus*, *Sus cebifrons*, *Sus celebensis*, *S. verrucosus*, and *P. africanus* [45–48].

### Quality control processing and mapping of next generation sequencing reads

To facilitate better reads mapping, three criteria of quality control were carried out using the NGSQC Toolkit (v2.30) [49]. First, the reads with adapter sequence were deleted. Second, the reads that contained more than 30% low-quality bases (quality value ≤20, or N bases) were discarded, and only paired reads were preserved. Finally, for each read, the low quality 3' ends with base quality scores <20 were trimmed. Next, the filtered paired-end reads were aligned individually to the Swine reference genome [50] using BWA v0.7.10 (BWA, RRID:SCR_010910) [14] with default parameters. We performed duplicate marking, base quality recalibration, duplicated reads removal, and mapping statistics (i.e., coverage of depth) by Picard v1.119 (Picard, RRID:SCR_006525), GATK v3.0 (GATK, RRID:SCR_001876), and SAMtools v1.3.1 (SAMTOOLS, RRID:SCR_002105) [51, 52]. Ultimately, these alignment files (BAM) were used directly for subsequent analyses, including SNV calling.

### Genome-wide variant calling and annotation

The aligned BAM files for 118 pigs were used for SNV detection on a population scale using SAMtools (v1.3.1) [51], including samtools, BCFtools, and vcfutils.pl scripts, respectively. The samtools mpileup command was run with the parameters “-u -C50 –DS -q20.” BCFtools and vcfutils.pl were run with the parameters “-evcgN” and “-d 20, –D 300” and they generated genotype calls in variant call format (VCF). In addition, an in-house Perl script was used to filter the QC parameters for each SNV VCF file, including Quality score = 999, MQ RMS mapping quality >20, DP >5, coverage >30%, and Alt-MAF >0.05. The SNVs were filtered out again by removing those within 5 bp of INDELs. The dbSNP database [53] was used to identify the novel genetic variations.

Finally, after filtering, the variants were processed for gene-based or region-based annotations using the ANNOVAR v2013–08-23 (ANNOVAR, RRID:SCR_012821) software [54], for which the corresponding gene annotation file was downloaded from the Ensembl database [55]. In the annotation step, SNVs were classified into eight categories based on their genomic locations, including exonic regions (synonymous, nonsynonymous, stop gain and stop loss), splicing sites, intronic regions, 5’ and 3’ UTRs, upstream and downstream regions, and intergenic regions.

### Phylogeny construction and PCA analysis

To better infer the genetic structure of pigs in our study, we constructed a phylogenetic tree using high-density SNV data with the following steps: First, we filtered all genotyped variants for the 29 pigs and converted these filtered variants (.vcf file) to PLINK format files (.ped and .map) using an in-house Perl script. Second, the identical by state (IBS) distance matrix between individuals was generated by the PLINK v1.07 (PLINK, RRID:SCR_001757) software [56] using the resulting   79,970,010 SNV sites. Finally, based on the distance matrix, the neighbor-joining tree was constructed by MEGA v6 (MEGA Software, RRID:SCR_000667) [57] and displayed with FigTree (v1.4.0) [58].

After filtering the SNVs from all pigs that had the same genotype, missing data, and a quality value <999, we performed the PCA with filtered SNVs using the GCTA software (v1.24.2) [59]. The genetic relationship matrix and the covariance matrix were inferred from the PLINK format files (.ped and .map) with the parameters “–make-grm, –pca 3.”Finally, we computed the eigenvectors based on the inferred covariance matrix and plotted the PCA biplot using R Packages.

### Analysis of population structure, LD decay, and demographic history

The construction of population structure used the program ADMIXTURE v1.3 (ADMIXTURE, RRID:SCR_001263) [21]. This program estimates the admixture proportions among different pigs using all   79,970,010 SNV high-density SNV data. Nine scenarios (ranging from K = 2 to K = 10) were selected for genetic clustering with the parameters: “major convergence criterion was 0.01.” LD levels for pig populations were assessed by genotype correlation coefficient (r2) between any two loci (within and between different chromosomes) using PLINK software [56]. The parameters were set as: “–blocks no-pheno-req –blocks-max-kb 10 000,”and then visualizations of LD decays among pig populations across the whole genome or chromosome were generated using R scripts.

The demographic analysis was conducted using the MSMC model as implemented in the MSMC (v0.1.0) software [22]. We set g = 5 and a rate of 1.25 × 10^−8^ mutations per generation to estimate the distribution of time and plotted the results using an in-house python script.

### Identifying the regions of Meishan pigs under selection

To detect the regions with significant selective signatures in Meishan pigs, we first calculated the Fst values to measure the population differentiation using a non-overlapping window approach with an in-house PERL script [60]. Then, we calculated the statistic$\ \ {d_i} = \mathop \sum \limits_{j \ne i} \frac{{F_{ST}^{ij} - E[ {F_{ST}^{ij}}]}}{{sd[ {F_{ST}^{ij}} ]}}\ $ for each SNV, where $E[ {F_{ST}^{ij}} ]$ and $sd[ {F_{ST}^{ij}} ]$ represent the expected value and standard deviation of Fst between breeds i and j calculated from 18 autosomes. Finally, ${{\rm{d}}_i}$ was averaged over SNVs in non-overlapping 100-kbwindows, and we empirically selected the significantly high Fst values the 1% right-tail as candidate signals in Meishan populations. Besides, to further measure the selection for Meishan pigs, CLR [61] was calculated for each population with non-overlapping 100-kbwindows using SweepFinder2 (v1.0) [62]. ΔCLR for Meishan pig was calculated by the formula: ΔCLR = CLR_Meishan_ − (CLR_Duroc_ + CLR_Tibetan_)/2. We also estimated allele frequencies of single SNV with a genome scan for each pig population and measured the absolute allele frequency difference (ΔAF) for comparing different populations. The ΔAF per SNV between the Meishan population and the other two populations was calculated using the formula: ΔAF = abs (AltAF_Meishan_—mean (AltAF_Duroc_ + AltAF_Tibetan_). The calculated ΔAF were binned in steps of 0.05 (i.e., 0–0.05, 0.05–0.10, etc. until 0.95–1.00) and displayed with a heatmap using “pheatmap” and “RcolorBrewer” R packages.

### Pairwise IBD detection between Meishan and Duroc population

A total of 67 individuals genotyped   17,792,807 SNV positions in the genome served as input for the IBD detection. The frequencies of shared haplotypes between Meishan and Duroc populations in different regions was estimated by per  10,000-bp bins using IBDLD (v3.37) [63]. The parameters were set as: “-plinkbf_int evolution -method GIBDLD -ploci 10 -nthreads 30 -step 0 -hiddenstates 3 -segment –length 10.”The nIBD between Meishan and Duroc populations was as follows: nIBD = cIBD/tIBD, where cIBD = count of all haplotypes IBD between Meishan and Drouc and tIBD = total pairwise comparisons between Meishan and Drouc. Known regions of Asian-derived introgression were downloaded from the supplementary information of previous study [7].

## Availability of data and material

A total 63 pig samples with 1,403.35Gbases were uploaded to NCBI with BioProject ID: PRJNA378496. Illumina paired-end sequences for the other 55 pigs used in this study were downloaded from NCBI with accession numbers ERP001813 and SRA065461. All supporting data, including VCF files, sweep regions, population genetic output files, and perl scripts, are available in the *GigaScience* repository, GigaDB [64].

## Additional files

Supplemental Table S1. Overall details of individual pigs.

Supplemental Table S2. 197 sweep regions with 300 candidate PCGs.

Supplemental Table S3. 197 sweep regions with 171 candidate lncRNAs.

Supplemental Table S4. Comparison of ΔAF per 0.05 bins within various functional regions.

Supplemental Table S5. Comparison of ΔAF per 0.05 bins within exonic regions.

Supplemental Table S6. 2999 Meishan-introgressed regions with the average nIBD value >0.10625.

Supplemental Table S7. 3.44 Mb Meishan-derived and Asian-derived regions.

Supplemental Table S8. Meishan-specific SNVs.

Supplemental Table S9. KEGG enrichment analyses.

## Abbreviations

ΔAF: allele frequency difference; CLR: composite likelihood ratio; HA: hyaluronan; IBD: identical by descent; kyBP: kilo years before present; LD: linkage disequilibrium; lncRNAs: long noncoding RNAs; MSMC: Markovian coalescent; *Ne*: effective population size; PCA: principal component analysis; PCGs: protein-coding genes; SNVs: single-nucleotide variants; UTR: untranslated regions.

## Ethics approval and consent to participate

The whole sample collection and treatment were conducted in strict accordance with the protocol approved by the Institutional Animal Care and Use Committee (IACUC) of China Agricultural University.

## Funding

This work was supported by the National High Technology Research and Development Program of China (863 Program 2013AA102503), the Program for Changjiang Scholar and Innovation Research Team in University (IRT1191), the National Natural Science Foundations of China (31661143013; 31790410), and Kunming Bureau of Science and Technology Key Program (09H130302).

## Competing interests

The authors declare that they have no competing interests.

## Authors' contributions

J-F.L. conceived and designed the experiments. P.Z. performed SNVs prediction and population analyses. W.F., J.Y., H.K., and H.D. contributed to computational analyses. X.Z. and H.K. collected samples and prepared for sequencing. P.Z., J-F.L. Y.Y., C.E., Z.W. and G.L. wrote and revised the paper. All authors read and approved the final manuscript.

## Supplementary Material

GIGA-D-18-00018_Original_Submission.pdf

GIGA-D-18-00018_Revision_1.pdf

Response_to_Reviewer_Comments_Original_Submission.pdf

Reviewer_1_Report_(Original_Submission) -- Hans Daetwyler 2/22/2018 Reviewed

Supplemental Tables
