# Supplementary material for: Evidence of evolutionary history and selective sweeps in the genome of Meishan pig reveals its genetic and phenotypic characterization
Source: Gigascience. 2018 May 15;7(5):giy058. doi: 10.1093/gigascience/giy058 (PMC6007440; doi:10.1093/gigascience/giy058)
Supplement: GIGA-D-18-00018_Original_Submission.pdf [file giy058_giga-d-18-00018_original_submission.pdf]

GigaScience

Evidence of Evolutionary History and Selective Sweep in the Genome of Meishan Pig Reveals its Genetic and Phenotypic Characterization

--Manuscript Draft--

**Manuscript Number:**	GIGA-D-18-00018
**Full Title:**	Evidence of Evolutionary History and Selective Sweep in the Genome of Meishan Pig Reveals its Genetic and Phenotypic Characterization
**Article Type:**	Research
**Funding Information:**	National High Technology Research and Development Program of China (863 Program 2013AA102503) — Prof. Jian-Feng Liu<br>Program for Changjiang Scholar and Innovation Research Team in University (IRT1191) — Prof. Jian-Feng Liu<br>the National Natural Science Foundations of China (31661146013) — Prof. Jian-Feng Liu<br>Kunming Bureau of Science and Technology Key Program (09H130302) — Prof. Jian-Feng Liu
**Abstract:**	Background: Meishan is a pig breed indigenous to China and famous for its high fecundity. The traits of Meishan strongly associated with its distinct evolutionary history and domestication. However, the genomic evidence linking the domestication of Meishan pigs with the unique features are still poorly understood. The goal of this study is to investigate the genomic signatures and evolutionary evidence related to phenotypic traits of Meishan by large-scale sequencing.<br>Results: We found the unique domestication of Meishan pigs happened at Taihu Basin area between the Majiabang and the Liangzhu culture, during which 300 protein-coding genes have undergone positive selection. Notably, the FoxO signaling pathway with significant enrichment signal and the harbored gene IGF1R were likely associated with high fertility of Meishan pig. Moreover, NFKB1 exhibited strong selective sweep signals and positively participated in hyaluronan biosynthesis as the key gene of KF-kB signaling, which might have resulted in the wrinkled skin and face of Meishan pig. Particularly, three population-specific synonymous single-nucleotide variants (SNVs) occurred in PYROXD1, MC1R, and FAM83G genes, of which T305C substitution in the MCIR gene explained the black coat in Meishan pig well. In addition, the shared haplotypes between Meishan and Duroc breeds confirmed the previous Asian-derived introgression and demonstrated the specific contribution of Meishan pigs.<br>Conclusions: The findings will help us explain the unique genetic and phenotypic characteristics of Meishan pigs, and offer a plausible method for the utilization of Meishan pigs as valuable genetic resources in pig breeding, and as the model animal for human wrinkled skin disease research.
**Corresponding Author:**	Jian-Feng Liu<br>China Agricultural University<br>China, Beijing CHINA
**Corresponding Author Secondary Information:**	
**Corresponding Author's Institution:**	China Agricultural University
**Corresponding Author's Secondary Institution:**	
**First Author:**	Pengju Zhao
**First Author Secondary Information:**	
**Order of Authors:**	Pengju Zhao<br>Ying Yu

Powered by Editorial Manager® and ProduXion Manager® from Aries Systems Corporation

	Wen Feng
	Heng Du
	Jian Yu
	Huimin Kang
	Xianrui Zheng
	Zhiquan Wang
	George E. Liu
	Catherine W. Ernst
	Jian-Feng Liu
**Order of Authors Secondary Information:**	
**Opposed Reviewers:**	
**Additional Information:**	
**Question**	**Response**
Are you submitting this manuscript to a special series or article collection?	No
**Experimental design and statistics**<br><br>Full details of the experimental design and statistical methods used should be given in the Methods section, as detailed in our Minimum Standards Reporting Checklist. Information essential to interpreting the data presented should be made available in the figure legends.<br><br>Have you included all the information requested in your manuscript?	Yes
**Resources**<br><br>A description of all resources used, including antibodies, cell lines, animals and software tools, with enough information to allow them to be uniquely identified, should be included in the Methods section. Authors are strongly encouraged to cite Research Resource Identifiers (RRIDs) for antibodies, model organisms and tools, where possible.<br><br>Have you included the information requested as detailed in our Minimum Standards Reporting Checklist?	Yes
**Availability of data and materials**<br><br>All datasets and code on which the	Yes

conclusions of the paper rely must be either included in your submission or deposited in publicly available repositories (where available and ethically appropriate), referencing such data using a unique identifier in the references and in the "Availability of Data and Materials" section of your manuscript.<br><br>Have you have met the above requirement as detailed in our Minimum Standards Reporting Checklist?	

Powered by Editorial Manager® and ProduXion Manager® from Aries Systems Corporation

Manuscript Click here to download Manuscript Manuscript.docx

Evidence of Evolutionary History and Selective Sweep in the Genome of Meishan Pig Reveals its Genetic and Phenotypic Characterization

Pengju Zhao[1], Ying Yu[1], Wen Feng[1], Heng Du[1], Jian Yu[1], Huimin Kang[1], Xianrui Zheng[1], Zhiquan Wang[2], George E. Liu[3], Catherine W. Ernst[4], Jian-Feng Liu[1#]

[1]National Engineering Laboratory for Animal Breeding; Key Laboratory of Animal Genetics, Breeding, and Reproduction, Ministry of Agriculture; College of Animal Science and Technology, China Agricultural University, Beijing, 100193, China.

[2]Department of Agricultural, Food & Nutritional Science, University of Alberta, Edmonton, T6G 2C8, Canada

[3]Animal Genomics and Improvement Laboratory, BARC, USDA-ARS, USA.

[4]Meat Animal Research Center, USDA-ARS, USA.

#Corresponding author:

Jian-Feng Liu, Ph.D.

China Agricultural University (West District)

College of Animal Science and Technology

Room 455

No.2 Yuanmingyuan West Road, Beijing 100193, China

Phone No.: 86-10-62731921

E-mail: liujf@cau.edu.cn

Email addresses:

Pengju Zhao: zhaopengju2014@gmail.com

Ying Yu: yuying@cau.edu.cn

Wen Feng: wfeng@cau.edu.cn

Heng Du: ahfydh@126.com

Jian Yu: yu_jian@cau.edu.cn

Huimin Kang: nongdaxiaokang@126.com

Xianrui Zheng: zxr07sk1@163.com

Zhiquan Wang: zhiquan.wang@ualberta.ca

George E. Liu: george.liu@ars.usda.gov

Catherine W. Ernst: ernstc@msu.edu.cn

Jian-Feng Liu: liujf@cau.edu.cn

Abstract

Background: Meishan is a pig breed indigenous to China and famous for its high fecundity. The traits of Meishan strongly associated with its distinct evolutionary history and domestication. However, the genomic evidence linking the domestication of Meishan pigs with the unique features are still poorly understood. The goal of this study is to investigate the genomic signatures and evolutionary evidence related to phenotypic traits of Meishan by large-scale sequencing.

Results: We found the unique domestication of Meishan pigs happened at Taihu Basin area between the Majiabang and the Liangzhu culture, during which 300 protein-coding genes have undergone positive selection. Notably, the FoxO signaling pathway with significant enrichment signal and the harbored gene *IGF1R* were likely associated with high fertility of Meishan pig. Moreover, *NFKB1* exhibited strong selective sweep signals and positively participated in hyaluronan biosynthesis as the key gene of KF-kB signaling, which might have resulted in the wrinkled skin and face of Meishan pig. Particularly, three population-specific synonymous single-nucleotide variants (SNVs) occurred in *PYROXD1*, *MC1R*, and *FAM83G* genes, of which T305C substitution in the *MCIR* gene explained the black coat in Meishan pig well. In addition, the shared haplotypes between Meishan and Duroc breeds confirmed the previous Asian-derived introgression and demonstrated the specific contribution of Meishan pigs.

Conclusions: The findings will help us explain the unique genetic and phenotypic characteristics of Meishan pigs, and offer a plausible method for the utilization of Meishan pigs as valuable genetic resources in pig breeding, and as the model animal for human wrinkled skin disease research.

Keywords: Large-scale sequencing; Meishan Pig; Selective Sweep; Fecundity; *IGF1R*; *MCIR*;

Background

The assembly of a Duroc pig (*Sus scrofa*) together with whole-genome sequencing from different pig populations provides a favorable opportunity for tracing the history of pig domestication and exploiting evidence of long-term gene flow and artificial selection [1]. Genome sequencing indicates that a deep phylogenetic split between European and Asian wild boars happened approximately one million years ago [1]. Subsequently, around 10,000 years ago, pigs were domesticated at multiple locations across Eurasia [2]. With sequencing costs dropping, several recent studies explored the origin, domestication, and evolutionary bottleneck of European and Asian native pigs [3-5]. Previous studies demonstrated that the distinct phenotypic characteristics between European and Asian pig breeds were due to the independent domestication of local wild boar populations in Asia and Europe. After the split between European and Asian pigs, the gene flow between Eurasian wild and domestic pig genomes, and human-mediated introgression have affected breed haplotypes [6, 7]. Especially, artificial selection affected behavior and morphology and led to different domestic traits in European and Asian pigs [6, 8, 9].

Among Asian pigs, the Meishan pig, named for the Chinese prefecture of Meishan, is well known as one of the most prolific breeds in the world. Besides its high fecundity, Meishan pigs have characteristics of early maturity, large drooping ears, and wrinkled black skin, which differ from that of the other pig breeds. The unique features of Meishan pig have received wide attention, and several studies have focused on the identification of genetic diversity and population structure of Meishan pig for unraveling potentially functional genes underlying its superior reproductive ability [10-12]. However, due to the high complexity of fecundity and related phenotypes, the genetic basis of Meishan pigs, particularly at the genomic level, remains largely unknown.

Following the idea that the formation of a domesticated species with typical characteristics was mainly caused by unique adaptive evolution to changing the climate and artificial selection [13], it can be presumed that the evolutionary history of Meishan owing to both natural and artificial selection could explain its specific biological characteristics. Therefore, to seek potential genomic evidence linking the domestication of Meishan pigs with their breed characters, we performed a large-scale sequencing and systematic comparison of 32 unrelated Meishan pigs with those of 86 other wild and domesticated pigs. We provided genomic evidence for the adaptive evolutionary history of the Meishan population and explored a suite of promising genes with Meishan-specific genomic variants and those

having undergone positive selection in Meishan genome. The findings herein will put insights into our understanding of genetic base determining the unique traits of Meishan pigs, and they laid a solid foundation for implementing the valuable resources of Meishan pigs into pig breeding and production as well as other relevant genetic studies.

.

Results

Genomic variant identification in Meishan pig breeds

To detect genome-wide variation in Meishan pig breeds, we performed whole-genome resequencing for 32 unrelated Meishan pigs aligned against the *Sus scrofa* 10.2 reference genome using BWA [14]; this generated a total of 732.76 Gb sequence data with above 8X mapped read depth on average (Table S1). Whole-genome SNVs were identified at the population level using the same variant detection pipelines and rigorous filtration criteria as set in our previous study [15]. A total of 9,789,671 SNVs with high quality were detected in the Meishan population (Fig. 1A), of which 18,366 SNVs were newly identified (not included in the dbSNP database [16]). These novel SNVs were expected to be present at lower frequencies or to be specific to the Meishan population, accounting for their not being previously detected (Fig. 1B).

Further annotation of these identified variants in Meishan population (Fig. 1C) revealed that the SNVs were most abundant in the intergenic regions (about 55.6%), followed by intronic, upstream and downstream, exonic, untranslated regions (UTR), and splicing site regions. Interestingly, we observed that more SNVs were located in the intronic regions of protein-coding genes (PCGs) than those of the long noncoding RNAs (lncRNAs); however, more SNVs were detected in the exonic regions of lncRNAs than those of the PCGs, suggesting that the selective pressure in the exonic regions of the PCGs was stronger than in the other functional regions. We also observed more genetic variations in the 3′-UTR (0.313% in the PCGs and 0.035% in the lncRNAs) than in the 5′-UTR (0.043% in the PCGs and 0.005% in the lncRNAs) in both PCGs and lncRNAs (Fig. 1C). This pattern is similar to that in the human genome [17]. With respect to exonic regions, we found a total of 51,985 potential functional genetic variations, including 34,170 synonymous SNVs, 17,625 non-synonymous SNVs, 155 stop-gain SNVs, and 35 stop-loss SNVs. These potential functional SNVs will provide valuable genetic resources for further exploring the genetic structure and selective signatures in the Meishan population.

Population diversity and demographic history

To infer the demographic history and the time of divergence of the Meishan population, we downloaded the sequence data of another 28 representative non-Meishan pig individuals, comprising 9 domestic pigs, 13 wild boars, 5 other *Sus* species, and one outgroup (*Phacochoerus africanus*) from

different geographical locations across three continents (Fig. 1D). A neighbor-joining tree of the pigs inferred from all SNVs was consistent with the results of previous studies and demonstrated strong clustering of pigs according to four major branches previously outlined on the basis of geographic and genetic classification [8, 18] (Fig. 1E); this might be because the pigs originated from very close geographical areas with domestication occurring under similar conditions.

The distribution of genetic distance (Fig. 2A) indicated that pigs from the same habitat were more likely to have similar genetic distance and the clearest clusters. However, further comparison of geographical distance and genetic distance among these non-Meishan breeds with the Meishan breed (Fig. 2B) revealed only weak correlation (*cor* = 0.41, *P* = 0.028). This suggests that the degree of genetic distance between different populations is determined not only by geographical isolation but also by the speciation time and human intervention. As shown in Fig. 2B, gene flow from Asian pig breeds to European breeds by means of artificial selection led to a relatively stronger genetic relationship between Meishan and European pigs (geographical distance/genetic distance = 85,336), especially domesticated European breeds (geographical distance/genetic distance = 90,758).

Based on genetic co-ancestry analyses [19], we partitioned all individuals into known groups by varying the number of presumed ancestral populations (Fig. 2C, K ranged from 2 to 10). When K was set to 4, four leading clusters were clearly observed—*Phacochoerus africanus and* other *Sus* species; Western domestic and wild pigs; Asian domestic and Tibetan wild boars; and Asian and Sumatran wild pigs. When K was set to 5, Tibetan wild boars could be separated from Asian domestic pigs. Interestingly, we also found that *Sus verrucosus* had more genetic commutation with *Sus scrofa* (Sumatran) than other *Sus* species, likely owing to recent human-mediated activities [20]. For values of K under 10, Meishan pigs were distinguishable from Asian domestic breeds and shared some genetic information with Jiangquhai pigs. Ternary principal component analysis (PCA) plots were also constructed with SNVs, revealing very similar patterns (Fig. 2D) to those identified by Admixture [21] with K=3.

As the unique genetic characters of Meishan pigs might be related to distinct divergence events, we further conducted a multiple sequentially Markovian coalescent (MSMC) analysis [22] for Meishan pigs and six other Chinese domestic populations as well as three Chinese wild boar population to infer historical changes in effective population size (*Ne*). A declining tendency for population size was detected in seven Chinese domestic pig populations through 7.2–4 kyBP (kilo years before present;

Fig. 2E); this period largely encompassed the post-glacial stage when temperatures appeared to be increasing and humans were moving into the modern period. In fact, the warm climate is beneficial to both development of human civilization and the domestication of pigs, showing that human-driven artificial selection may result in a “bottleneck” in the evolution of different domesticated breeds. Most interestingly, unlike the other six Chinese domestic pig populations, Meishan pigs showed a later bottleneck, with the occurrence of a marked bottleneck 4,000–5,000 years ago (red line, Fig. 2E), reflecting that Meishan breeds more likely undergo a unique domestication process. Exactly, in the Taihu Basin area, three cultures were recorded during this period (7–4 kyBP)—the Majiabang Culture (7–6 kyBP), Songze Culture (6–5 kyBP), and Liangzhu Culture (5–4 kyBP). Archaeological evidence indicates the presence of pigs at the site of the Taihu basin around 7,040 years ago [23]. We accordingly inferred that the unique domestication of Meishan pigs in Taihu Basin area started from the Majiabang Culture, and continuously developed as late as the Liangzhu Culture.

Population structure and selection sweeps

To in-depth mine the genomic evidence contributing to Meishan pig’s breed features, we further compared the genome signatures of Meishan pigs at the population level with two other typical pig breeds with the characters greatly differing from Meishan pigs, *i.e.*, 30 Tibetan wild boars as representatives of Asian wild boar population, and 35 Duroc pigs representing European domesticated breeds. Genetic distinctiveness among these pig populations reflected the pattern of isolation by their adaptation/environment (Fig. 3A). Intra-population genetic distance for each breed was considerably smaller than the inter-population genetic distance between the different breeds (Fig. 3B). Furthermore, the intra-population genetic distance of domesticated pig breeds was lower than that of Tibetan wild boars. This demonstrated that artificial selection tends to reduce genetic diversity, and commercial breeds have undergone stronger artificial selection than have local breeds. The impact of artificial selection was also reflected in the genome linkage disequilibrium (LD) levels in each population (Fig. 3C), reflecting that artificial selection can facilitate the increase of LD within a population [24]. PCA also revealed a similar pattern (Fig. 3D); the Meishan population was shaped in a tight cluster and clearly separated from other populations.

Based on the population-scale genetic differences between Meishan and other pig breeds, we speculated that there should exist specific genome signals in the Meishan population arising from long-term artificial and positive natural selection during domestication. To further identify the genomic

locations of these selective sweeps in Meishan pigs, we calculated the genome-wide statistic d_i [25] for the Meishan to Duroc and Tibetan wild boar populations. Focusing on the regions at the top 1% of the d_i empirical distribution (Fig. 4A), we identified 197 significant regions (d_i > 1.672) harboring 300 candidate PCGs (204 functional annotated genes) and 171 lncRNA genes (Table S2-3). Among these genes, composite likelihood ratio (CLR) tests [26] revealed that a major proportion of PCGs (57.33%) and lncRNAs (53.22%) also fell into regions of selective sweeps with stronger positive selection signals in Meishan than in other breeds (Fig. 4B); these regions commonly determined by both CLR and d_i statistic may be potentially related to selection during the domestication of Meishan pigs.

As the highly differentiated SNVs across populations more readily occurred in the vicinity of the region under selection [27], we further compared the alternate allele frequency (the introduced new allele) of all identified SNVs in the Meishan population with those in the other two populations. Quantitative distribution of SNVs in Meishan and the other two populations were surveyed by grouping all SNVs with frequencies harbored in the corresponding interval in steps of 0.05 (*i.e.*, 0–0.05, 0.05–0.10, *etc*. until 0.95–1.00) (Fig. 4C). We accordingly calculated the absolute allele frequency difference [$\Delta AF = abs\ \{AltAF_{Meishan} - mean\ (AltAF_{Duroc} + AltAF_{Tibetan})\}$] between the Meishan population and the other two populations to assess the potential selective sweeps of Meishan breeds. We determined that 56,305 breed-specific SNVs were merely fixed in the Meishan population (ΔAF=1). Significant enrichment was noted for high-ΔAF SNVs (> 0.8) within the identified sweep regions, particularly the overlapping regions identified using both CLR and d_i methods (Fig. 4D), reflecting the fact that the highly differentiated population SNVs were actually associated with artificial and natural selection. Further comparison of ΔAF per 0.05 bins within various functional regions (exonic, intronic, UTRs, and so on) (Table S4–5) revealed significant enrichment for low-ΔAF SNVs ($0.1<\Delta AF<0.35$, x^2 test, $P < 9.98 \times 10^{-7}$) in exonic regions, but significant enrichment for high-ΔAF SNVs ($0.55<\Delta AF<1$, x^2 test, $P < 0.0014$) in intronic regions. Notably, for the SNVs in exonic regions, we observed a significant excess of synonymous SNVs within different ΔAF bins ($0.3<\Delta AF<0.8$, x^2 test, $P < 0.0012$), but non-synonymous SNVs were largely enriched in the low-ΔAF bin ($0<\Delta AF<0.15$, x^2 test, $P < 0.0012$). The results supported the supposition that most of the genetic changes during domestication are concentrated in the regulatory region rather than the coding regions [27].

Meishan-derived introgression in European pigs

Meishan pigs, as the major Chinese pig breed, contributed considerably to improving commercial production in European pig breeds during the Industrial Revolution [7]. Here we identified the present region of introgressed Meishan haplotypes in European domestic pigs (Duroc) using the pairwise identical by descent (IBD) method [7]. We calculated the normalized IBD (nIBD) for each 10,000-bp bin in the pig genome to estimate the extent of introgression events, and the top 5% of nIBD regions were regarded as evidence of Meishan-derived introgression into European pigs.

We observed a total of 12,272 bins (122.7 Mb) (Fig. 5A) with an average nIBD value > 0.10625 and finally merged 2,999 Meishan-introgressed regions with the length range from 10 to 1,430 kb (Table S6). Interestingly, of these, 3.44 Mb Meishan-derived regions (n=121) have been identified as Asian-derived introgression in European pigs by Groenen *et al* [7] (Table S7), Remarkably, the presence of the two longest consecutive regions (in chromosomes 8 and 9) of Asian-derived introgression was confirmed in Meishan haplotypes (Fig. 5B). We also found introgression signals (nIBD > 0.2) of Meishan pig on Chromosome 9 near (~190 kb) the Aryl Hydrocarbon Receptor (*AHR*) gene, which is known to be associated with female fertility and increased litter size in previous studies [7, 28]. Besides, we also observed some meat quality-related genes such as Spalt Like Transcription Factor 1 (*SAL1*) and Malic Enzyme 1 (*ME1*), which also shared more haplotypes with Asian domesticated pigs than with European wild boars. These findings demonstrate the contribution of Meishan pigs to the formation of Asian haplotypes in European pigs during the Industrial Revolution.

In addition to these shared Asian pig originated haplotypes, some regions of Meishan-derived haplotypes also provided potential evidence of Meishan fertile introgression. For instance, these regions contain the Gonadotropin Releasing Hormone Receptor (*GNRHR*) and Gonadotropin Releasing Hormone 1 (*GNRH1*) genes, both of which are associated with hypogonadotropic hypogonadism and play an important role in reproduction (Fig. 5C). Particularly in the *GNRHR* gene, some genetic variations (SNVs) have been associated with litter size in goats [29]. Therefore, the results further proved that Meishan pig introgressions play a vital role in European haplotypes, especially sow fertility, and these novel Meishan-derived introgressions could provide new insight into the artificial selection of modern European pig breeds.

Characterization of candidate genes underlying breed feature of Meishan pigs

Through comparison of gene frequency between Meishan and the other two representative breeds, we identified a total of 280 candidate Meishan-specific SNVs of exonic regions with the criterion of $AF_{Meishan} > 95\%$ and $AF_{non\text{-}Meishan} < 5\%$ (Table S8). These SNVs fell into the regions of 244 PCGs (132 functionally annotated genes). Of note, 114 of the 244 PCGs (52 functionally annotated genes) appeared at a higher evolutionary rate in Meishan breeds, and their coding structures were changed by 125 large-effect mutations—123 nonsynonymous and 2 stop-gain SNVs. In particular, of these 125 large-effect mutations, 3 perfectly fixed synonymous SNVs ($\Delta AF = 1$) occurred respectively in *PYROXD1*, *MC1R*, and *FAM83G* genes. Intriguingly, these three genes had been shown in studies on humans to play an important role in the development of skeletal muscle [30], skin [31, 32], and bone [33]. Particularly, we found a missense SNV (exon1:c.T305C:p.L102P; rs45434630 in the dbSNP database) within the Melanocortin 1 receptor (*MC1R*) gene that generated a Leu-to-Pro substitution, leading to a change in the *MC1R* protein conformation space (Fig. 6A). The *MC1R* gene is mainly expressed in the melanocytes of hair follicles and controls melanogenesis, which had been proved to be associated with black (dominant E^D) coat color pattern in pigs [31]. Therefore, the identification of this nonsynonymous mutation in *MC1R* helps us to better explain the black coat of the Meishan breed.

Aside from the aforementioned potentially fixed genes, we further collected all 300 PCGs (204 functionally annotated genes) with significant positive selection signals identified in the Meishan genome for follow-up pathway analyses. KEGG enrichment analyses detected a total of 34 pathways harboring 120 of these 204 annotated PCGs ($P< 0.05$; Table S9). Intriguingly, the identified FoxO signaling pathway exhibited the strongest enrichment statistical signal (corrected P =0.022), with five positive selection related genes involved (*ATM*, *CSNK1E*, *CCNB1*, *GABARAP*, and *IGF1R*). The FoxO signaling pathway has regulated ovarian prostaglandins, which are critical for reproduction [34]. We further focused on the most promising gene, insulin-like growth factor 1 receptor (*IGF1R*) involved in the FoxO signaling pathway Previous studies have reported *IGF1R* to be crucial for female fertility as it participates in steroidogenesis, follicle survival, and fertility in female mice [35, 36]. Although there was no direct evidence that polymorphisms for *IGF1R* were associated with litter size, numerous *IGF1R* mutations had been proved to affect late prenatal and early postnatal growth restriction, perinatal growth velocity, and diminutive body size [37, 38]. Proper litter size of piglets ensures a higher number of surviving offspring [39]; this is borne out by the large litter size of Meishan pigs. As expected, strong selective sweep signals (d_i value = 1.80; CLR > 965) and 17 high-ΔAF SNVs

(ΔAF > 0.8; including 3 synonymous SNVs and 14 intronic SNVs) were noted in the *IGF1R* gene region of the Meishan genome (Fig. 6B). We inferred therefrom that the *IGF1R* gene and corresponding FoxO signaling pathway may be promising candidates for prolificacy-related positive selection in the domestication of the Meishan population.

Another typical phenotypic characteristic of Meishan breed is the wrinkled face and skin, which is also observed in Shar-Pei dogs and human patients with folding and thickening of the skin [40] (Fig. 6C). This abnormality of cutaneous tissue is mainly due to anomalies in hyaluronan (HA) metabolism; the high activity of HA synthase increases the activity of dermal fibroblasts and gradually leads to the formation of wrinkled skin [41]. Previous studies have used Shar-Pei dogs as research model animals to successfully identify the candidate gene (hyaluronan synthase 2; *HAS2*) responsible for the skin wrinkles in Shar-Peis [42], but no direct evidence exists for the role of this gene in the extreme thickening of the human skin so far. In contrast to the results of the Shar-Pei study, we did not detect *HAS2* (LOC100152156) in the selection region of the Meishan breed. However, we observed strong selective sweep signals (*di* value = 1.77; CLR > 89) and 43 high-ΔAF SNVs (ΔAF > 0.8; including 1 synonymous SNV, 1 UTR3, 1 downstream, and 40 intronic SNVs) in the Nuclear Factor Kappa B Subunit 1 (*NFKB1*) gene region (Fig. 6D), which, as the key gene involved in KF-kB signaling pathway, has been proven relevant to the positive regulation of HA biosynthesis [43, 44]. These results may better explain why wrinkled skin largely occurs on the face and neck in Meishan pigs. Together with the finding in Shar-Peis, we speculated both *HAS2* and *NFKB1* can act as candicate genes likely associated with the incidence of wrinkled skin in humans since these two genes were reported palying key roles in HA biosynthesis.

Discussion

This study provides the first comprehensive large-scale re-sequencing and survey for the Meishan pig breed with the highest prolificacy known in the world. Our results, as well as the downloaded re-sequencing data, will support future in-depth analyses on population genetics, demographic history, genomic selection, introgression, and breed-specific genetic variations in pigs. The identification of selective sweep regions, introgression regions, and breed-specific genetic variations associated with superior fecundity in Meishan pigs may contribute toward molecular marker-based breeding for improved pig reproduction.

The present study revealed that Meishan pigs share a similar, but not identical, genetic background with other Asian pigs; this finding is consistent with the fact that there is a domestication bottleneck caused by human-driven artificial selection and distinct domestication approximately 4,000 ~ 5,000 years ago. This period in the Taihu Basin overlapped with the Liangzhu culture, when the climate was warm and dry, rice agriculture was developing, and the human population was rapidly increasing. The unique climate of the Liangzhu civilization and land conditions of the Taihu basin had a considerable lasting effect on the domestication of the Meishan breed and prompted the improvement of prolificacy traits.

We identified 244 Meishan-specific fixed genes and 300 PCGs undergoing positive selection. Of note, the FoxO signaling pathway with significant enrichment signal and the harbored gene *IGF1R* may be the most promising genomic evidence explaining the high fertility of Meishan pigs. We found that *NFKB1* exhibited strong selective sweep signals and as the key gene in the of KF-kB signaling pathway it plays an important role in HA biosynthesis, which is more likely induced wrinkled skin and face of Meishan pig. These findings will help explain the unique phenotype characteristics of Meishan pig and provide new insights into the causes of infertility and skin with extreme thickening and folding in humans.

We provide new evidence that Meishan pigs greatly contributed to the improvement of commercial traits in European pig breeds during the Industrial Revolution. Notably, 3.44 M regions were also supported by Asian-derived introgression, including the two longest consecutive regions (chromosome 8 and 9) and the *AHR* haplotype. Moreover, we supplemented some novel Meishan-derived introgression regions and genes (*GNRH1*, *GNRHR*, *etc*); this information will likely provide

insights into the artificial selection of modern European pig breeds. Meishan pigs are one of the main Asian domesticated pigs that were introduced into Europe; however, they still retained several of their breed-specific genetic variations. About 58 known PCGs were found to be influenced greatly by breed-specific genetic variations in our study. For instance, the black coat in Meishan pig can be explained by the presence of the missense SNV with T305C substitution in the *MCIR* gene. Therefore, these findings will be valuable for further studies on the Meishan breed.

Our findings will facilitate the explanation of the unique characteristics of Meishan pigs and offer a plausible method for their utilization as valuable genetic resources in pig breeding. Obviously, these fertility-related markers could be used in selection to increase fertility in pigs, so that the increasing number of live-born piglets. The potential role of *NFKB1* gene as a new candidate biomarker that helps us to improve our understanding of human patients with folding and thickening of the skin. It is worth noting that three Meishan-specific synonymous SNVs detected in *PYROXD1*, *MC1R*, and *FAM83G* genes respectively, exhibiting further research value for Meishan-specific phenotypic traits. *MC1R* has been proved as the key amino acid mutation that leads to the black coat of the Meishan breed. Although few studies of *PYROXD1* and *FAM83G* genes on pig genome, it has been associated with the development of human skeletal muscle and bone. Therefore, it's worth expecting that these *PYROXD1* and *FAM83G* genes may shed new insights into slow growth traits of Meishan pig in a future study.

Conclusions

In summary, the increased knowledge of Meishan phenotype-related genes helps to improve our understanding of the underlying biological mechanisms contributing to fertility, black coat, wrinkled skin, even growth traits in pigs, as well as other mammals including humans.

Methods and Materials

Samples collection and sequencing

We sequenced a total of 63 samples (32 Meishan pigs form Kunshan city of Jiangsu province and 31 Durocs form Yancheng city of Jiangsu province) in this study (Table S1). We used the Qiagen DNeasy Tissue kit (Qiagen, Germany) to extract genomic DNA from pig ear tissue, and controlled the quality and integrity of DNA based on agarose gel electrophoresis and A260/280 ratio. All qualified genomic DNA was sequenced using an Illumina HiSeq 2000 sequencing system at Novogene (Beijing, China). The Illumina DNA libraries (Paired-end, 2 x 125 bp) were constructed for 63 pig samples and 1403.35G bases were generated.

In summary, a total of 118 pigs were selected from 10 pig domesticated breeds, 13 wild boars and 5 other *Su*s species and a genus of wild pig from different geographical locations, including 32 Meishan pigs, 35 Durocs, 30 Tibetan wild boars, a Yorkshire, a Landrace, a European wild boar (Meinweg, the Netherlands) , a European wild boar (Veluwe, the Netherlands) , a European wild boar (Switzerland), a Japan wild boar, a Asian wild boar (South China), a Asian wild boar (North China), a Asian wild boar (Southest China), a Min pig, a Jangquhai, a Bamaxiang, a Rongchang pig, a Diannan small-ear pigs, a Daweizi pig, a *Sumatran*, a *Sus barbatus*, a *Sus cebifrons*, a *Sus celebensis*, a *Sus verrucosus* and a *Phacochoerus africanus* [45-48].

NGS reads QC processing and mapping

To facilitate better reads mapping, three criteria of quality control (QC) were carried out by NGSQC Toolkit [49]. First, the reads with adapter sequence were deleted. Second, the reads which contained more than 30 percent low-quality bases (quality value ≤ 20, or N bases) were discarded, and only paired reads were preserved. Finally, for each read, the low quality 3' end with base quality scores lower than 20 were trimmed. Next, the filtered paired-end reads were aligned individually to the Swine reference genome (*Sus scrofa10.2*: ftp://ftp.ensembl.org/pub/release-67/fasta/sus_scrofa/dna/) using BWA[14] with default parameters. We performed duplicate marking, base quality recalibration, duplicated reads removal and mapping statistics (i.e. coverage of depth) by Picard, GATK, and SAMtools [50, 51]. Ultimately, these alignment files (bam) were used directly for subsequent analyses, including SNV calling.

Genome-wide variant calling and annotation

The aligned BAM files for 118 pigs were used for SNV detection on a population scale using SAMtools [50], including samtools, bcftools and vcfutils.pl scripts, respectively. The samtools mpileup command was run with the parameters "-u -C50 –DS -q20". BCFtools and vcfutils.pl were run with the parameters "-evcgN" and "-d 20, –D 300" and generated genotype calls in Variant Call Format (VCF). In addition, an in-house Perl script was used to filter the QC parameters for each SNV VCF file, including Quality score equal 999, MQ RMS mapping quality >20, DP > 5, coverage > 30%, Alt-MAF >0.05. Besides, the SNVs have further filtered out again by removing those within 5 bp of INDELs. The dbSNP database (ftp://ftp.ncbi.nih.gov/snp/organisms/pig_9823/VCF/) was used to identify the novel genetic variations.

Finally, the variants after filtering were processed for gene-based or region-based annotations using the ANNOVAR software [52], for which the corresponding gene annotation file was downloaded from the Ensembl database (http://hgdownload.soe.ucsc.edu/goldenPath/susScr3/database/ensGene.txt.gz). In the annotation step, SNVs were classified into eight categories based on their genome locations, including exonic regions (synonymous, nonsynonymous, stop gain and stop loss), splicing sites, intronic regions, 5' and 3' UTRs, upstream and downstream regions, and intergenic regions.

Phylogeny construction and PCA analysis

To better infer the genetic structure of pigs in our study, we constructed the phylogenetic tree using high-density SNV data with the following steps: First, we filtered all genotyped variants for the 29 pigs and converted these filtered variants (.vcf file) to PLINK format files (.ped and .map) using an in-house Perl script. Second, the IBS distance matrix between individuals was generated by the PLINK software [53] using the resulting 79,970,010 SNV sites. Finally, based on the distance matrix, the neighbor-joining (NJ) tree was constructed by MEGA (v6) [54] and displayed by FigTree (v1.4.0)[55].

After filtering the SNVs from all pigs that had the same genotype, missing data, and a quality value lower than 999, we performed the PCA with filtered SNVs using the GCTA software (v1.24.2) [56]. The genetic relationship matrix (GRM) and the covariance matrix were inferred from the PLINK format files (.ped and .map) with the parameters "--make-grm, --pca 3". Finally, we computed the eigenvectors based on the inferred covariance matrix and plotted the PCA biplot using R packages.

Analysis of population structure, LD decay, and Demographic history

The construction of population structure used the program ADMIXTURE [21]. It estimates the admixture proportions among different pigs using all 79,970,010 SNV high-density SNV data. Nine scenarios (ranging from K = 2 to K = 10) were selected for genetic clustering with the parameters: “major convergence criterion was 0.01”. Levels of linkage disequilibrium (LD) for pig populations were assessed by genotype correlation coefficient (r2) between any two loci (within and between different chromosomes) using PLINK (Version1.90) software [53]. The parameters were set as: “--blocks no-pheno-req --blocks-max-kb 10000”, and then visualization of LD decay among pig populations across the whole genome or chromosome were generated using R scripts.

The demographic analysis was conducted using the multiple sequential Markovian coalescent (MSMC) model as implemented in the MSMC software [22]. We set g = 5 and a rate of $1.25\text{x}10^{-8}$ mutations per generation to estimate the distribution of time and plotted the results using an in-house python script.

Identifying the regions of Meishan pigs under selection

To detect the regions with significant selective signatures of Meishan pigs, we first calculated the Fst values to measure the population differentiation using non-overlapping window approach with an in-house PERL script [57]. And then we calculated the statistic $d_i = \sum_{j \neq i} \frac{F_{ST}^{ij} - E[F_{ST}^{ij}]}{sd[F_{ST}^{ij}]}$ for each SNV, where $E[F_{ST}^{ij}]$ and $sd[F_{ST}^{ij}]$ represent the expected value and standard deviation of Fst between breeds i and j calculated from 18 autosomes. Finally, d_i was averaged over SNVs in non-overlapping 100-Kb windows and we empirically selected the significantly high Fst values the 1% right-tail as candidate signals in Meishan populations. Besides, to further measure the selection for Meishan pigs, CLR [58] was calculated for each population with non-overlapping 100-Kb windows using SweepFinder2 [59]. ΔCLR for Meishan pig was calculated by the formula: $\Delta CLR = CLR_{Meishan} - (CLR_{Duroc} + CLR_{Tibetan})/2$.

We also estimated allele frequencies of single SNV with a genome scan for each pig population and measured the absolute allele frequency difference (ΔAF) for comparing different populations. the ΔAF per SNV between Meishan population and other two populations was calculated using the formula: $\Delta AF = abs\ (AltAF_{Meishan} – mean\ (AltAF_{Duroc} + AltAF_{Tibetan})$. The calculated ΔAF were binned in steps of 0.05 (i.e. 0–0.05, 0.05–0.10, etc. until 0.95-1.00) and displayed with a heatmap using R packages.

Pairwise IBD detection between Meishan and Duroc population

A total of 67 individuals genotyped 17,792,807 SNV positions in the genome served as input for the IBD detection. the frequency of shared haplotypes between Meishan and Duroc population in different regions were estimated by per 10,000 bp bins using IBDLD (v3.37) [60]. The parameters were set as: "-plinkbf_int evolution -method GIBDLD -ploci 10 -nthreads 30 -step 0 -hiddenstates 3 -segment --length 10". The Normalized IBD between Meishan and Duroc population as follows: nIBD=cIBD/tIBD, where cIBD=count of all haplotypes IBD between Meishan and Drouc and tIBD =total pairwise comparisons between Meishan and Drouc. Known regions of Asian-derived introgression were download from the supplementary information of previous study [7].

List of abbreviations

ΔAF: allele frequency difference; CLR: composite likelihood ratio; GRM: genetic relationship matrix; HA: hyaluronan; IBD: identical by descent; kyBP: kilo years before present; LD: linkage disequilibrium; LncRNAs: Long noncoding RNAs; MSMC: Markovian coalescent; *Ne*: effective population size; NJ: neighbor-joining; PCA: principal component analysis; PCGs: protein-coding genes; SNVs: single-nucleotide variants; UTR: untranslated regions;

Declarations

Ethics approval and consent to participate

The whole sample collection and treatment were conducted in strict accordance with the protocol approved by the Institutional Animal Care and Use Committee (IACUC) of China Agricultural Univers ity.

Availability of data and material

Totally 63 pig samples with 1403.35G bases were upload to NCBI with BioProject ID: PRJNA378496. Illumina paired-end sequences for other 55 pigs used in this study were downloaded from NCBI with accession numbers: ERP001813 and SRA065461.

Competing interests

The authors declare that they have no competing interests.

Funding

This work was supported by the National High Technology Research and Development Program of China (863 Program 2013AA102503), the Program for Changjiang Scholar and Innovation Research

Team in University (IRT1191), the National Natural Science Foundations of China (31661146013), and Kunming Bureau of Science and Technology Key Program (09H130302).

Authors' contributions

J-F.L. conceived and designed the experiments. P.Z. performed SNVs prediction and population analyses. W.F., J.Y., H.K., and H.D. contributed to computational analyses. X.Z. and H.K. collected samples and prepared for sequencing. P.Z., J-F.L. Y.Y., C.E., Z.W. and G.L. wrote and revised the paper. All authors read and approved the final manuscript.

Acknowledgements

We thank Editage company for offering professional English language editing to this study.

Figure Legends

Figure 1. SNV characteristics of Meishan pig and the geographic and genetic relationship for 29 representative pig breeds.

A. Nucleotide diversities of Meishan pig breeds and their presence within the dbSNP database. Bar plots represent the number of SNVs. Pie charts show the percent of Meishan SNVs within the dbSNP database.

B. The relationship between known and novel variants with various allele frequencies. Bar plots represent the percentage (%) of genetic variations within various allele frequencies.

C. Gene annotation of genetic variations. Bar plots represent the number of genetic variations ($\log_{10}$) within various functional regions.

D. Geographic origin of 29 analyzed pig breeds. Twenty-nine analyzed subspecies from the four main geographic groups were collected from Europe and America (n = 6; purple circles), Africa (n = 1; black circle), Asia (n = 17; red circles), and the Southeast Asia (n = 5; blue circles).

E. Neighbor-joining tree constructed from SNV data among 29 subspecies.

Figure 2. Population diversity and demographic history of Meishan pigs.

A. The heatmap inferring the genetic relationship using SNV data from 29 subspecies. The heatmap was used to reveal the genetic distance between pairwise subspecies.

B. Scatter diagram showing the relationship between geographic and genetic distance among 29 subspecies. The X-axis represents the genetic distance and Y-axis represents the geographic distance.

C. ADMIXTURE analysis showing clustering of samples from 29 subspecies within K groups. K refers to the number of presumed ancestral groups.

D. PCA plot with SNV data. Different colors represent different subspecies.

E. Demographic history of Meishan and other Chinese domestic and wild pigs. Generation time (g) = 5 year and transversion mutation rate (u) = 1.25×10^{-8} mutations per bp per generation.

Figure 3. Genetic relationships and population structure among three pig populations.

A. Neighbor-joining phylogenetic tree of three pig populations. The genetic distance is measured by SNV data from Meishan, Duroc, and Tibetan wild boar populations.

B. Frequency distribution of genetic distance among three pig populations. The X-axis represents the genetic distance and Y-axis represents frequency.

C. Length distribution of LD block among three pig populations. Red, purple, and blue bar represent

Meishan, Duroc, and Tibetan wild boar populations, respectively.

D. PCA plot for three pig populations with SNV data. Different colors represent different pig populations.

Figure 4. Selection sweeps of Meishan population.

A. Definition of sweep regions for Meishan population. The X-axis represents the D_i value and Y-axis represents CLR value. Red circles mean sweep regions for Meishan population.

B. The plot of CLR values among three pig populations. Part 1 shows the genome-wide distribution of ΔCLR; Part 2–3 represent the genome-wide distribution of CLR signal values for Meishan, Duroc, and Tibetan wild boar respectively.

C. Quantitative distribution of SNVs between Meishan and other population within various bins. The heatmap showing the number of SNVs ($\log_{10}$) with a bin in steps of 0.05.

D. A number of high ΔAF SNVs within various specific regions. The overlap regions represent the overlap between di-method and CLR-method based regions.

Figure 5. Meishan-derived introgression in European pigs.

A. The map of Meishan-derived introgression in European pigs. The red bars represent the Meishan-derived introgression regions within chromosome 1–18.

B. Two confirmed longest Asian-derived regions. The black bars represent the Meishan-derived introgression, and the red bars represent the Asian-derived introgression.

C. *GNRH1* and *GNRHR* genes in Meishan-derived introgression region. Line charts represent the nIBD value distribution within Meishan-derived introgression region. The gray bar indicates the position of *GNRH1* and *GNRHR* genes.

Figure 6. Specific genes with strong selective sweep signals in Meishan pigs.

A. Prediction of protein conformation space for *MC1R*. Structure of Amino acids coded by exon 8 of porcine *MC1R*, as predicted by SWISS-MODEL.

B. Candidate gene *IGF1R* for prolificacy in Meishan. D_i values, CLR values, and ΔCLR values are plotted surrounding *IGF1R* gene. The bottom part showing the gene structure and all high ΔAF SNVs within a gene. The gray bar indicates the position of the *IGF1R* gene.

C. Comparison of phenotype between the wrinkled and unwrinkled skin.

D. Candidate gene *NFKB1* for wrinkled skin and face of Meishan pig and the labelling is same to Figure 6B.

References

1. Groenen MA, Archibald AL, Uenishi H, Tuggle CK, Takeuchi Y, Rothschild MF, et al. Analyses of pig genomes provide insight into porcine demography and evolution. Nature. 2012;491 7424:393-8. doi:10.1038/nature11622.
2. Larson G, Dobney K, Albarella U, Fang M, Matisoo-Smith E, Robins J, et al. Worldwide phylogeography of wild boar reveals multiple centers of pig domestication. Science. 2005;307 5715:1618-21. doi:10.1126/science.1106927.
3. Li M, Chen L, Tian S, Lin Y, Tang Q, Zhou X, et al. Comprehensive variation discovery and recovery of missing sequence in the pig genome using multiple de novo assemblies. Genome Res. 2016; doi:10.1101/gr.207456.116.
4. Bosse M, Megens HJ, Madsen O, Crooijmans RP, Ryder OA, Austerlitz F, et al. Using genome-wide measures of coancestry to maintain diversity and fitness in endangered and domestic pig populations. Genome Res. 2015;25 7:970-81. doi:10.1101/gr.187039.114.
5. Li M, Tian S, Yeung CK, Meng X, Tang Q, Niu L, et al. Whole-genome sequencing of Berkshire (European native pig) provides insights into its origin and domestication. Sci Rep. 2014;4:4678. doi:10.1038/srep04678.
6. Frantz LA, Schraiber JG, Madsen O, Megens HJ, Cagan A, Bosse M, et al. Evidence of long-term gene flow and selection during domestication from analyses of Eurasian wild and domestic pig genomes. Nat Genet. 2015;47 10:1141-8. doi:10.1038/ng.3394.
7. Bosse M, Megens HJ, Frantz LA, Madsen O, Larson G, Paudel Y, et al. Genomic analysis reveals selection for Asian genes in European pigs following human-mediated introgression. Nat Commun. 2014;5:4392. doi:10.1038/ncomms5392.
8. Ai H, Fang X, Yang B, Huang Z, Chen H, Mao L, et al. Adaptation and possible ancient interspecies introgression in pigs identified by whole-genome sequencing. Nat Genet. 2015;47 3:217-25. doi:10.1038/ng.3199.
9. Bosse M, Megens HJ, Madsen O, Paudel Y, Frantz LA, Schook LB, et al. Regions of homozygosity in the porcine genome: consequence of demography and the recombination landscape. PLoS Genet. 2012;8 11:e1003100. doi:10.1371/journal.pgen.1003100.
10. Wang Z, Chen Q, Liao R, Zhang Z, Zhang X, Liu X, et al. Genome-wide genetic variation discovery in Chinese Taihu pig breeds using next generation sequencing. Animal Genetics. 2017;48 1:38-47. doi:10.1111/age.12465.
11. Wang Z, Chen Q, Yang Y, Liao R, Zhao J, Zhang Z, et al. Genetic diversity and population structure of six Chinese indigenous pig breeds in the Taihu Lake region revealed by sequencing data. Anim Genet. 2015;46 6:697-701. doi:10.1111/age.12349.
12. Xiao Q, Zhang Z, Sun H, Yang H, Xue M, Liu X, et al. Genetic variation and genetic structure of five Chinese indigenous pig populations in Jiangsu Province revealed by sequencing data. Anim Genet. 2017; doi:10.1111/age.12560.
13. Olson-Manning CF, Wagner MR and Mitchell-Olds T. Adaptive evolution: evaluating empirical support for theoretical predictions. Nat Rev Genet. 2012;13 12:867-77. doi:10.1038/nrg3322.

14. Li H and Durbin R. Fast and accurate short read alignment with Burrows-Wheeler transform. Bioinformatics. 2009;25 14:1754-60. doi:10.1093/bioinformatics/btp324.
15. Kang H, Wang H, Fan Z, Zhao P, Khan A, Yin Z, et al. Resequencing diverse Chinese indigenous breeds to enrich the map of genomic variations in swine. Genomics. 2015;106 5:286-94. doi:10.1016/j.ygeno.2015.08.002.
16. Sherry ST, Ward MH, Kholodov M, Baker J, Phan L, Smigielski EM, et al. dbSNP: the NCBI database of genetic variation. Nucleic Acids Res. 2001;29 1:308-11.
17. Zhao Z, Fu YX, Hewett-Emmett D and Boerwinkle E. Investigating single nucleotide polymorphism (SNP) density in the human genome and its implications for molecular evolution. Gene. 2003;312:207-13.
18. Groenen MA. A decade of pig genome sequencing: a window on pig domestication and evolution. Genet Sel Evol. 2016;48:23. doi:10.1186/s12711-016-0204-2.
19. Alexander DH, Novembre J and Lange K. Fast model-based estimation of ancestry in unrelated individuals. Genome Res. 2009;19 9:1655-64. doi:10.1101/gr.094052.109.
20. Frantz LA, Madsen O, Megens HJ, Groenen MA and Lohse K. Testing models of speciation from genome sequences: divergence and asymmetric admixture in Island South-East Asian Sus species during the Plio-Pleistocene climatic fluctuations. Mol Ecol. 2014;23 22:5566-74. doi:10.1111/mec.12958.
21. Falush D, Wirth T, Linz B, Pritchard JK, Stephens M, Kidd M, et al. Traces of human migrations in Helicobacter pylori populations. Science. 2003;299 5612:1582-5. doi:10.1126/science.1080857.
22. Schiffels S and Durbin R. Inferring human population size and separation history from multiple genome sequences. Nat Genet. 2014;46 8:919-25. doi:10.1038/ng.3015.
23. Qian H, Wang H, Xie Z, Huang Z, Shi G and Jiang S. Discovery of Impact Breccias in the Western of Taihu Lake in Jangsu Province, China: New Evidence for an Impact Origin. Meteorit Planet Sci. 2010;45:A167-A.
24. O'Brien AMP, Utsunomiya YT, Meszaros G, Bickhart DM, Liu GE, Van Tassell CP, et al. Assessing signatures of selection through variation in linkage disequilibrium between taurine and indicine cattle. Genetics Selection Evolution. 2014;46 doi:Artn 1910.1186/1297-9686-46-19.
25. Akey JM, Ruhe AL, Akey DT, Wong AK, Connelly CF, Madeoy J, et al. Tracking footprints of artificial selection in the dog genome. Proc Natl Acad Sci U S A. 2010;107 3:1160-5. doi:10.1073/pnas.0909918107.
26. Pavlidis P, Zivkovic D, Stamatakis A and Alachiotis N. SweeD: likelihood-based detection of selective sweeps in thousands of genomes. Mol Biol Evol. 2013;30 9:2224-34. doi:10.1093/molbev/mst112.
27. Carneiro M, Rubin CJ, Di Palma F, Albert FW, Alfoldi J, Martinez Barrio A, et al. Rabbit genome analysis reveals a polygenic basis for phenotypic change during domestication. Science. 2014;345 6200:1074-9. doi:10.1126/science.1253714.
28. Hernandez-Ochoa I, Karman BN and Flaws JA. The role of the aryl hydrocarbon receptor in the female reproductive system. Biochem Pharmacol. 2009;77 4:547-59. doi:10.1016/j.bcp.2008.09.037.
29. Li G, Wu HP, Fu MZ and Zhou ZQ. Novel single nucleotide polymorphisms of

GnRHR gene and their association with litter size in goats. Arch Tierzucht. 2011;54 6:618-24.

30. O'Grady GL, Best HA, Sztal TE, Schartner V, Sanjuan-Vazquez M, Donkervoort S, et al. Variants in the Oxidoreductase PYROXD1 Cause Early-Onset Myopathy with Internalized Nuclei and Myofibrillar Disorganization. Am J Hum Genet. 2016;99 5:1086-105. doi:10.1016/j.ajhg.2016.09.005.
31. Kijas JM, Wales R, Tornsten A, Chardon P, Moller M and Andersson L. Melanocortin receptor 1 (MC1R) mutations and coat color in pigs. Genetics. 1998;150 3:1177-85.
32. Dun G, Li X, Cao H, Zhou R and Li L. Variations of melanocortin receptor 1 (MC1R) gene in three pig breeds. J Genet Genomics. 2007;34 9:777-82. doi:10.1016/S1673-8527(07)60088-5.
33. Vogt J, Dingwell KS, Herhaus L, Gourlay R, Macartney T, Campbell D, et al. Protein associated with SMAD1 (PAWS1/FAM83G) is a substrate for type I bone morphogenetic protein receptors and modulates bone morphogenetic protein signalling. Open Biol. 2014;4:130210. doi:10.1098/rsob.130210.
34. Edmonds JW, Prasain JK, Dorand D, Yang Y, Hoang HD, Vibbert J, et al. Insulin/FOXO signaling regulates ovarian prostaglandins critical for reproduction. Dev Cell. 2010;19 6:858-71. doi:10.1016/j.devcel.2010.11.005.
35. Baumgarten SC, Armouti M, Ko C and Stocco C. IGF1R Expression in Ovarian Granulosa Cells Is Essential for Steroidogenesis, Follicle Survival, and Fertility in Female Mice. Endocrinology. 2017;158 7:2309-18. doi:10.1210/en.2017-00146.
36. Law NC and Hunzicker-Dunn ME. Insulin Receptor Substrate 1, the Hub Linking Follicle-stimulating Hormone to Phosphatidylinositol 3-Kinase Activation. J Biol Chem. 2016;291 9:4547-60. doi:10.1074/jbc.M115.698761.
37. Abuzzahab MJ, Schneider A, Goddard A, Grigorescu F, Lautier C, Keller E, et al. IGF-I receptor mutations resulting in intrauterine and postnatal growth retardation. N Engl J Med. 2003;349 23:2211-22. doi:10.1056/NEJMoa010107.
38. Harris RA, Tardif SD, Vinar T, Wildman DE, Rutherford JN, Rogers J, et al. Evolutionary genetics and implications of small size and twinning in callitrichine primates. Proc Natl Acad Sci U S A. 2014;111 4:1467-72. doi:10.1073/pnas.1316037111.
39. Andersen IL, Naevdal E and Boe KE. Maternal investment, sibling competition, and offspring survival with increasing litter size and parity in pigs (Sus scrofa). Behav Ecol Sociobiol. 2011;65 6:1159-67. doi:10.1007/s00265-010-1128-4.
40. Olsson M, Meadows JR, Truve K, Rosengren Pielberg G, Puppo F, Mauceli E, et al. A novel unstable duplication upstream of HAS2 predisposes to a breed-defining skin phenotype and a periodic fever syndrome in Chinese Shar-Pei dogs. PLoS Genet. 2011;7 3:e1001332. doi:10.1371/journal.pgen.1001332.
41. Ramsden CA, Bankier A, Brown TJ, Cowen PSJ, Frost GI, McCallum DD, et al. A new disorder of hyaluronan metabolism associated with generalized folding and thickening of the skin. J Pediatr-Us. 2000;136 1:62-8. doi:Doi 10.1016/S0022-3476(00)90051-9.
42. Docampo MJ, Zanna G, Fondevila D, Cabrera J, Lopez-Iglesias C, Carvalho A, et al. Increased HAS2-driven hyaluronic acid synthesis in shar-pei dogs with hereditary cutaneous hyaluronosis (mucinosis). Veterinary Dermatology. 2011;22 6:535-45. doi:10.1111/j.1365-3164.2011.00986.x.

43. Vigetti D, Genasetti A, Karousou E, Viola M, Moretto P, Clerici M, et al. Proinflammatory Cytokines Induce Hyaluronan Synthesis and Monocyte Adhesion in Human Endothelial Cells through Hyaluronan Synthase 2 (HAS2) and the Nuclear Factor-kappa B (NF-kappa B) Pathway. Journal of Biological Chemistry. 2010;285 32:24639-45. doi:10.1074/jbc.M110.134536.
44. Hanabayashi M, Takahashi N, Sobue Y, Hirabara S, Ishiguro N and Kojima T. Hyaluronan Oligosaccharides Induce MMP-1 and-3 via Transcriptional Activation of NF-kappa B and p38 MAPK in Rheumatoid Synovial Fibroblasts. Plos One. 2016;11 8 doi:ARTN e016187510.1371/journal.pone.0161875.
45. Frantz LA, Schraiber JG, Madsen O, Megens HJ, Bosse M, Paudel Y, et al. Genome sequencing reveals fine scale diversification and reticulation history during speciation in Sus. Genome Biol. 2013;14 9:R107. doi:10.1186/gb-2013-14-9-r107.
46. Rubin CJ, Megens HJ, Martinez Barrio A, Maqbool K, Sayyab S, Schwochow D, et al. Strong signatures of selection in the domestic pig genome. Proc Natl Acad Sci U S A. 2012;109 48:19529-36. doi:10.1073/pnas.1217149109.
47. Zhao P, Li J, Kang H, Wang H, Fan Z, Yin Z, et al. Structural Variant Detection by Large-scale Sequencing Reveals New Evolutionary Evidence on Breed Divergence between Chinese and European Pigs. Sci Rep. 2016;6:18501. doi:10.1038/srep18501.
48. Li M, Tian S, Jin L, Zhou G, Li Y, Zhang Y, et al. Genomic analyses identify distinct patterns of selection in domesticated pigs and Tibetan wild boars. Nat Genet. 2013;45 12:1431-8. doi:10.1038/ng.2811.
49. Patel RK and Jain M. NGS QC Toolkit: a toolkit for quality control of next generation sequencing data. PLoS One. 2012;7 2:e30619. doi:10.1371/journal.pone.0030619.
50. Li H, Handsaker B, Wysoker A, Fennell T, Ruan J, Homer N, et al. The Sequence Alignment/Map format and SAMtools. Bioinformatics. 2009;25 16:2078-9. doi:10.1093/bioinformatics/btp352.
51. McKenna A, Hanna M, Banks E, Sivachenko A, Cibulskis K, Kernytsky A, et al. The Genome Analysis Toolkit: a MapReduce framework for analyzing next-generation DNA sequencing data. Genome Res. 2010;20 9:1297-303. doi:10.1101/gr.107524.110.
52. Wang K, Li M and Hakonarson H. ANNOVAR: functional annotation of genetic variants from high-throughput sequencing data. Nucleic Acids Res. 2010;38 16:e164. doi:10.1093/nar/gkq603.
53. Purcell S, Neale B, Todd-Brown K, Thomas L, Ferreira MA, Bender D, et al. PLINK: a tool set for whole-genome association and population-based linkage analyses. Am J Hum Genet. 2007;81 3:559-75. doi:10.1086/519795.
54. Kumar S, Nei M, Dudley J and Tamura K. MEGA: a biologist-centric software for evolutionary analysis of DNA and protein sequences. Brief Bioinform. 2008;9 4:299-306. doi:10.1093/bib/bbn017.
55. Drummond AJ, Suchard MA, Xie D and Rambaut A. Bayesian phylogenetics with BEAUti and the BEAST 1.7. Mol Biol Evol. 2012;29 8:1969-73. doi:10.1093/molbev/mss075.
56. Yang J, Lee SH, Goddard ME and Visscher PM. GCTA: a tool for genome-wide complex trait analysis. Am J Hum Genet. 2011;88 1:76-82.

doi:10.1016/j.ajhg.2010.11.011.

57. Holsinger KE and Weir BS. Genetics in geographically structured populations: defining, estimating and interpreting F(ST). Nat Rev Genet. 2009;10 9:639-50. doi:10.1038/nrg2611.

58. Jensen JD, Kim Y, DuMont VB, Aquadro CF and Bustamante CD. Distinguishing between selective sweeps and demography using DNA polymorphism data. Genetics. 2005;170 3:1401-10. doi:10.1534/genetics.104.038224.

59. DeGiorgio M, Huber CD, Hubisz MJ, Hellmann I and Nielsen R. SWEEPFINDER2: increased sensitivity, robustness and flexibility. Bioinformatics. 2016;32 12:1895-7. doi:10.1093/bioinformatics/btw051.

60. Han L and Abney M. Identity by Descent Estimation With Dense Genome-Wide Genotype Data. Genet Epidemiol. 2011;35 6:557-67. doi:10.1002/gepi.20606.

Figure 1

Click here to download Figure Fig. 1.tif

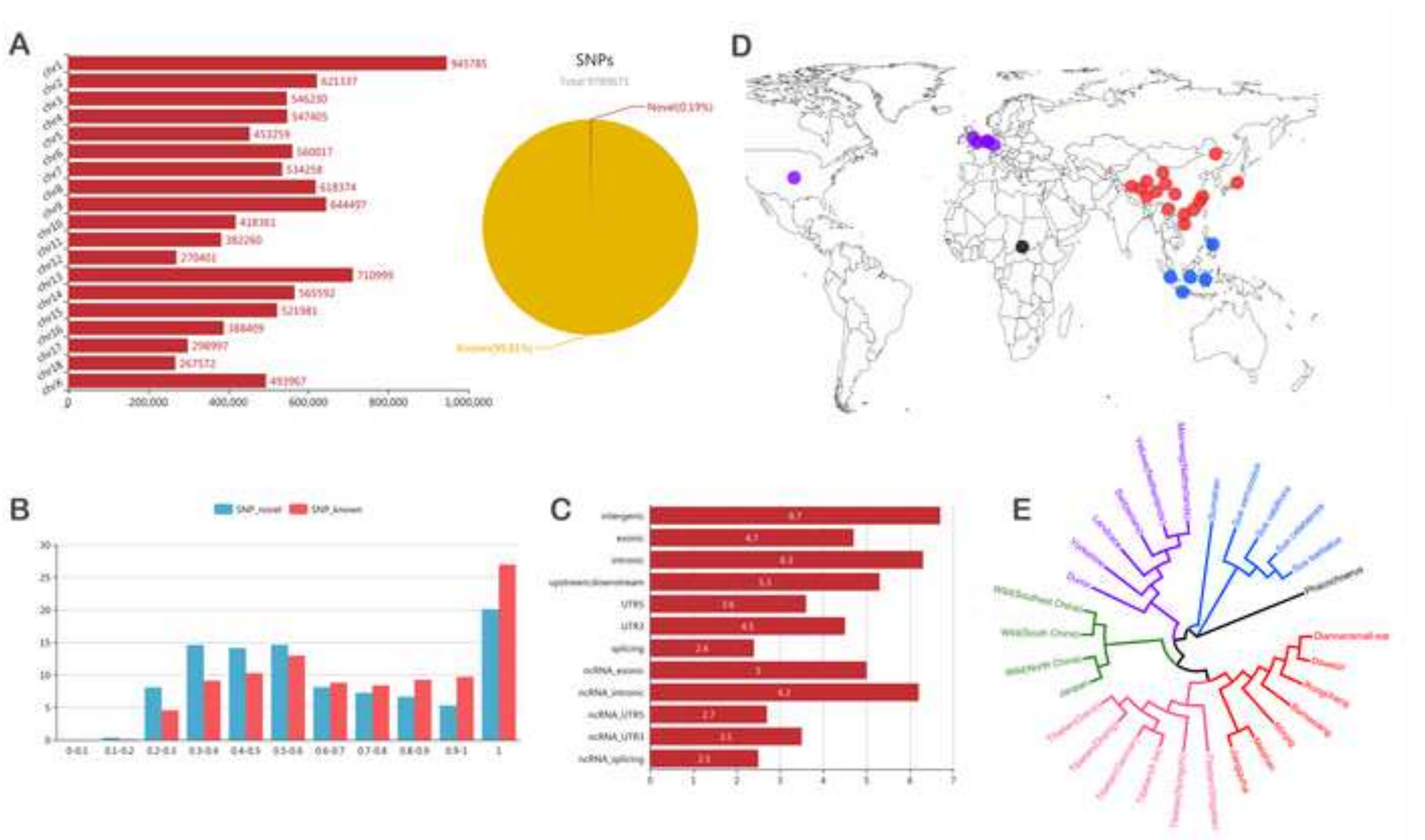

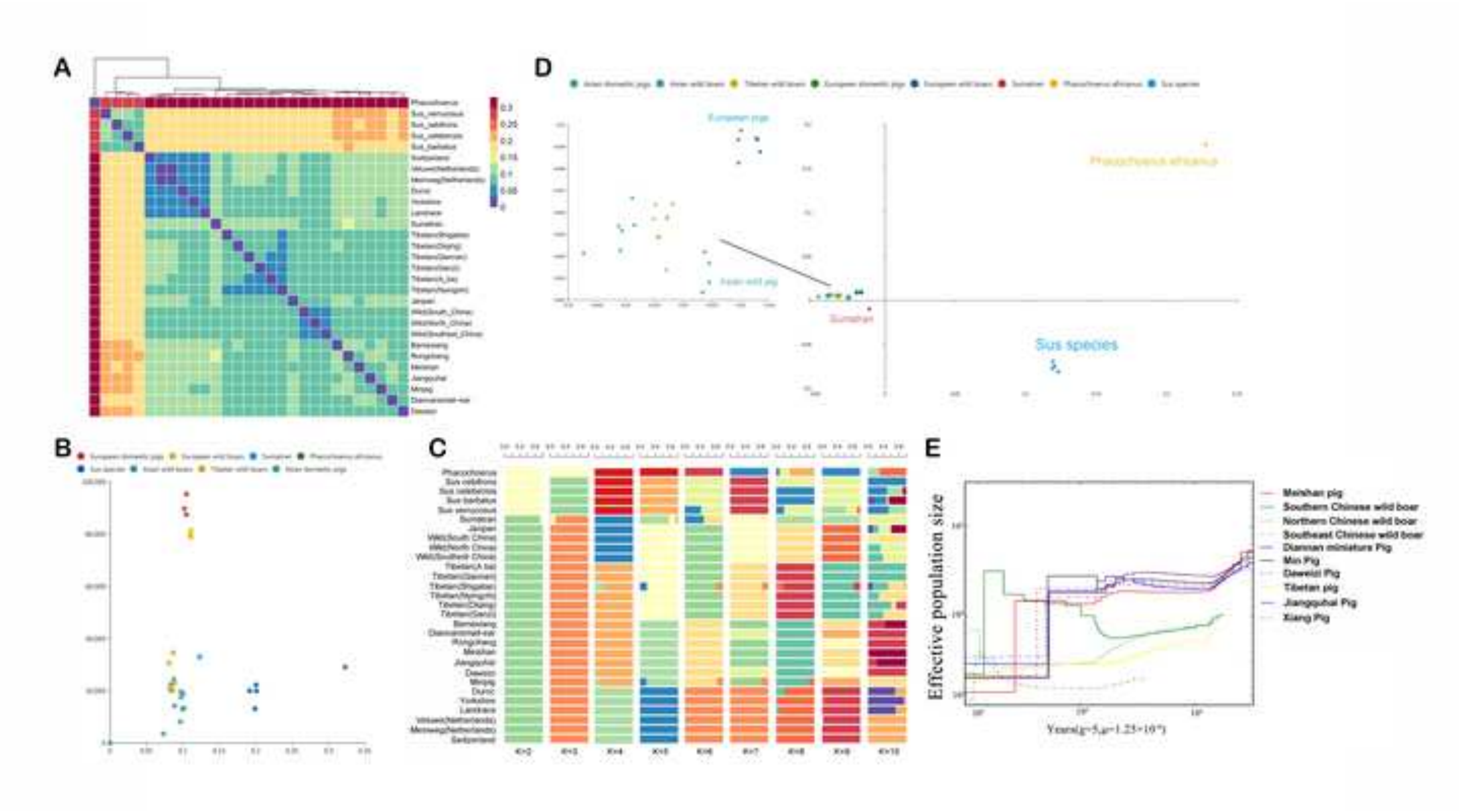
A
B
C
D
E
European pigs
Asian wild pig
Sumatran
Phacochoerus africanus
Sus species
Meishan pig
Southern Chinese wild boar
Northern Chinese wild boar
Southeast Chinese wild boar
Dianmian miniature Pig
Min Pig
Daweizi Pig
Tibetan pig
Jiangquhai Pig
Xiang Pig
Effective population size

Figure 3

Click here to download Figure Fig. 3.tif

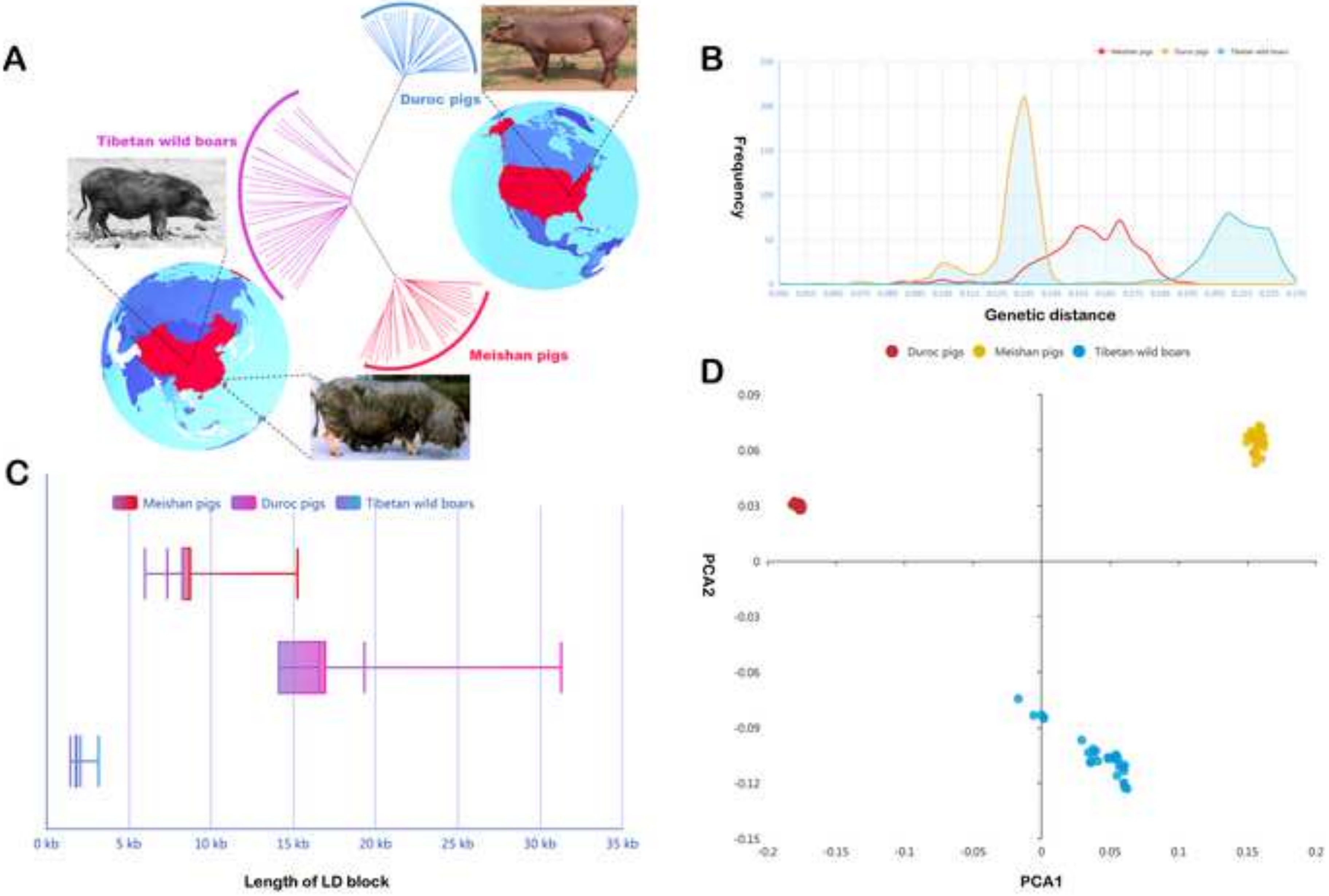

Figure 4

Click here to download Figure Fig. 4.tif

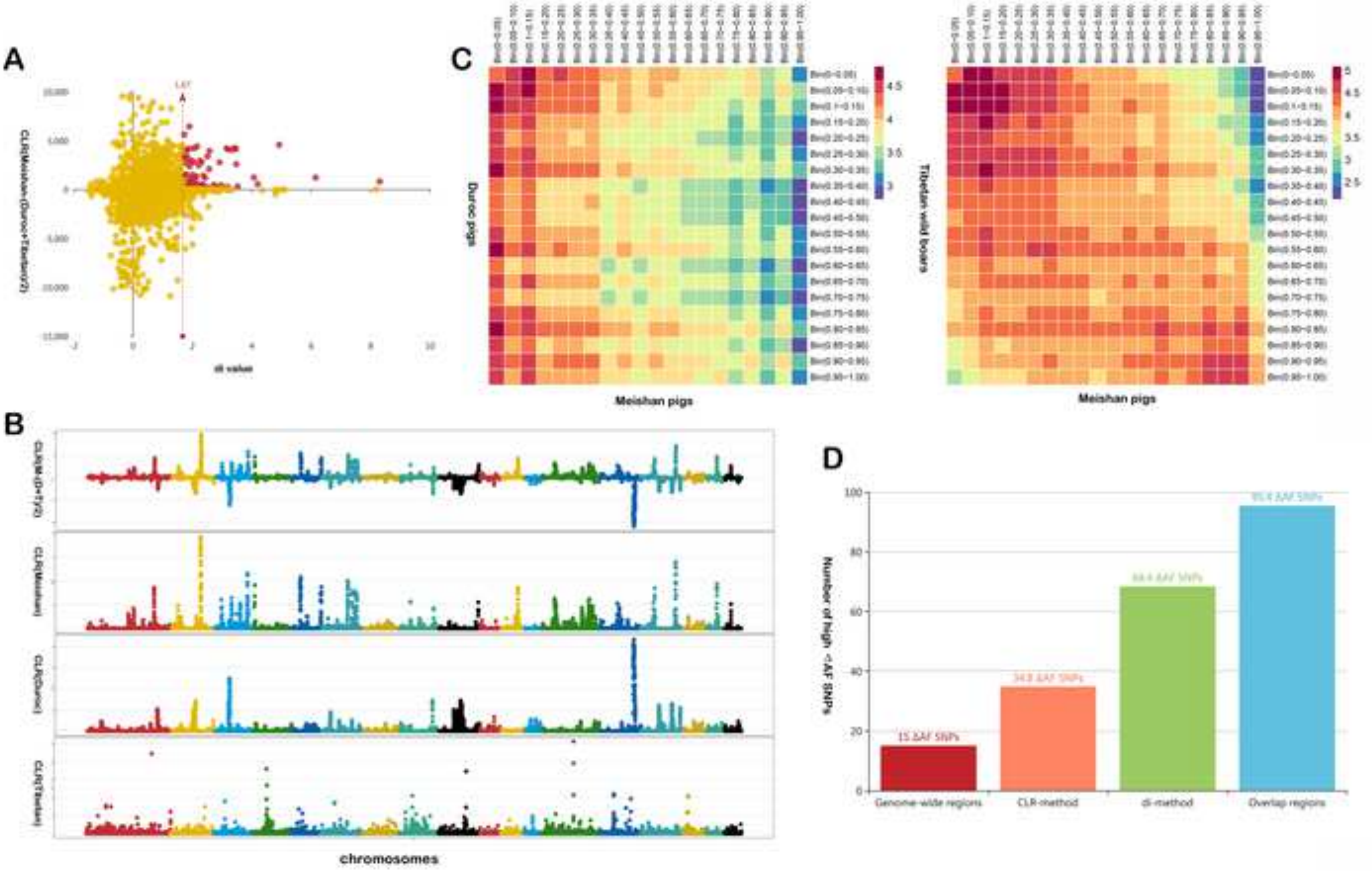

Figure 5

Click here to download Figure Fig. 5.tif

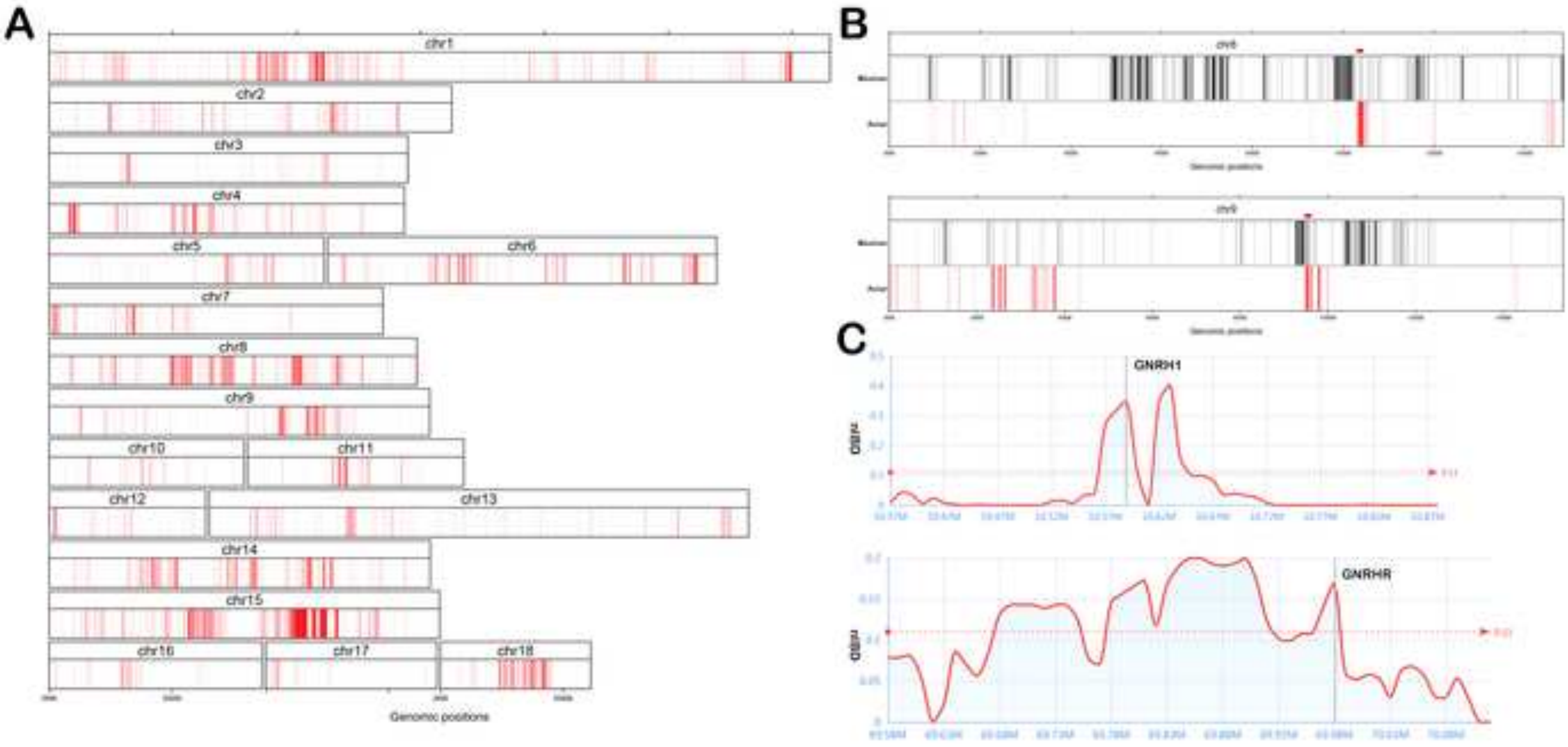

Figure 6 Click here to download Figure Fig. 6.tif

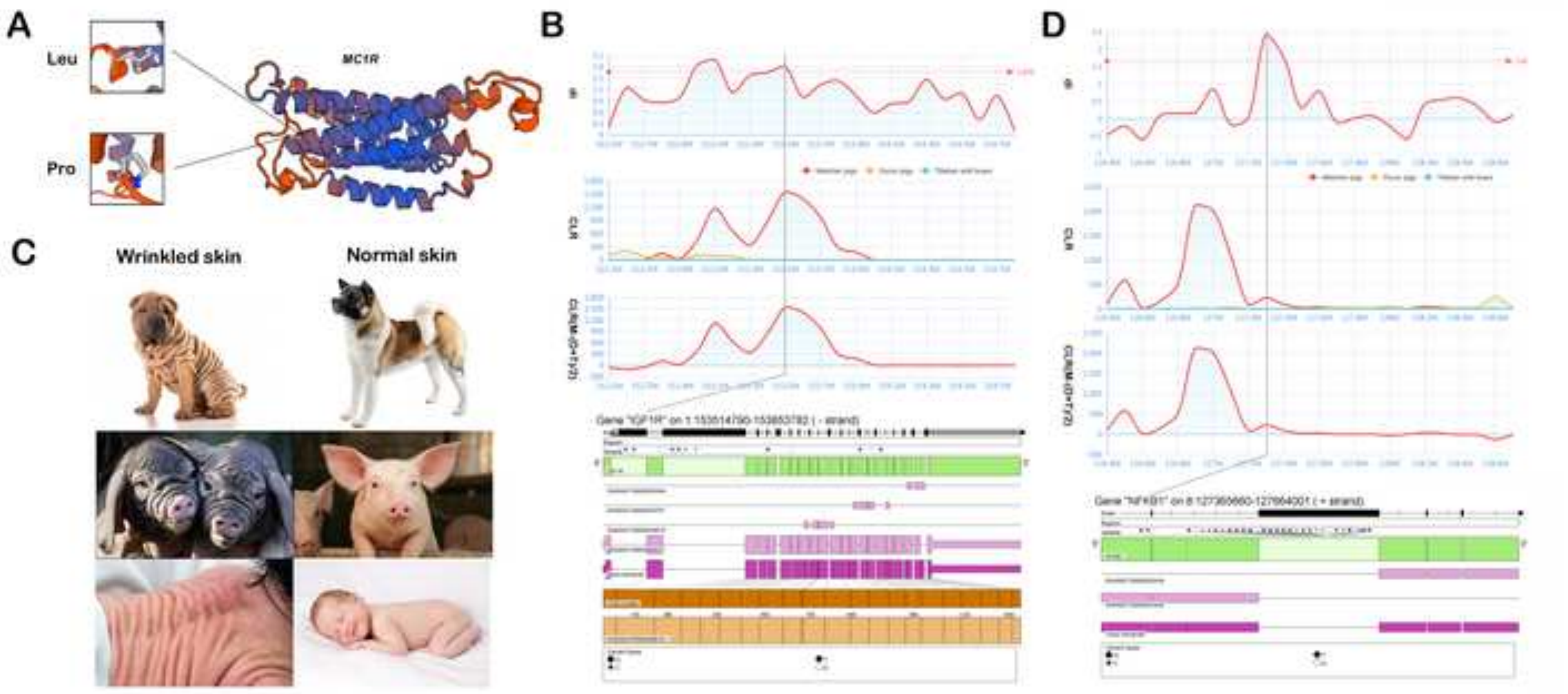

Click here to access/download
Supplementary Material
Supplemental_Tables.xlsx

Dear editor and reviewers,

Enclosed is an original article entitled "*Evidence of Evolutionary History and Selective Sweep in the Genome of Meishan Pig Reveals its Genetic and Phenotypic Characterization*" by Pengju Zhao, Ying Yu, Wen Feng, Heng Du, Jian Yu, Huimin Kang, Xianrui Zheng, George E. Liu, Catherine W. Ernst and Jian-Feng Liu.

Thank you very much for giving us an opportunity to resubmit our revised manuscript to ***GigaScience.*** According to all concerns pointed out by three reviewers in our previous manuscript (GIGA-D-17-00044), we seriously considered and substantially addressed all comments in the revision.

In our resubmitted work, we performed a large-scale sequencing and systematic analyses of Meishan pig genome to identify genomic signatures related to its unique genetic and phenotypic traits. We identified two leading protein-coding genes, *IGF1R* and *NFKB1*, that had undergone positive selection and identified strong selective sweep signals. Significantly, *IGF1R* was involved in the FoxO signaling pathway, which was likely associated with high fertility of Meishan pig. *NFKB1 was inferred* to be associated with the wrinkled skin and face of Meishan pig based on its functional mutation in other species. In addition, a substitution in the *MCIR* gene explained the black coat in Meishan pig well. Our work makes a significant contribution to the literature regarding the breed feature of Meishan.

The findings herein will facilitate the explanation of the unique characteristics of Meishan pigs, and offer a plausible method for their utilization as valuable genetic resources in pig breeding. Therefore, we believed that this paper will be of interest to animal breeders, geneticists, and evolutionary biologists, who constitute the readership of your journal.

Therefore, we would like to resubmit it to you for consideration of publication in ***GigaScience.*** This article is not considered elsewhere for publication in part or in full, in any language. All authors have reviewed and approved this final version of the manuscript. There is no conflict of interest in our submission.

We thank you very much for handling our manuscript!

Below are all comments of the previous manuscript (GIGA-D-17-00044) from three reviewers and the corresponding revised strategies in our new version of the manuscript.

Best regards,

Jian-Feng Liu,Ph.D.
China Agricultural University

Reviewer reports:
Reviewer #1: Review report

GigaScience
Manuscript Number: GIGA-D-17-00044

The manuscript entitled "Domestication bottleneck and genetic divergence revealed in Meishan pigs by population-scale sequencing" by Zhao et al. describes an ambitious effort to examine evolutionary history of a Chinese pig breed, Meishan, and in a comparative examination with other breeds to identify genes controlling unique fecundity characteristics of the breed. They sequenced a total of 119 animals from 11 pig breeds and 12 wild boars and reported > 73 millions of SNPs and ~9 millions of Indels. The authors ran an handful of common programs to explore population structure / phylogeny and apply metrics such as Fst, ROH, Tajima's D and OMEGA for scanning the genome for selective sweeps representing differentiated genes between prolific Meishan and other breeds as candidates of reproductive performance. Eventually, they present a panel of genes overlapping differentiated CNVs or SNPs and report a Meishan-specific missense mutation as a candidate for prolificacy.

Response: Thanks for your generally positive comments on our works

The topic is interesting and manuscript uses a valuable data set. There are issues need to be addressed where I listed some below.
Line 150: "Reduced global genetic variations of MS pigs": variations or variation?

Response: The issue was no longer involved in the new manuscript. Thanks for your guidance.

Line 150: "Reduced global genetic variations of MS pigs". Nucleotide diversity is the right metric to quantify/compare genetic variation. Therefore, the statement "The fewest InDels and fewest 157 SNPs were detected in MS pigs compared to the other population" is not a precise and efficient here.

Response: Thank you for comments. Sorry for our inaccuracy statement and we had corrected this viewpoint in the new manuscript

Line 160 and 164: what the terms "redundant" and non-redundant" are implying for?

Response: Thanks for your comments. The term “redundant” SNP represents the SNPs were identified more than once in multiple populations. The term “non-redundant” SNP represents the unique SNP in the specific population.

Line 189: What the term "very similar" implies for, in the following sentence.

"Our analysis found that the major clusters of the pig populations deduced by SNPs and InDels are very similar".

Response: Sorry for the unclear description herein. In our revision, we merely focus on SNP data analyses to avoid potential confusions. Thanks!

Line 211: This sentence must be re-worded. "Therefore, we further inferred domestication and human-driven

artificial selection may be the reason for a "bottleneck" in the evolutionary process of different domesticated breeds."

Response: Thank you for your suggestion, we made the corresponding change in the new manuscript at page 8, Lines 2-7.

Line 229: "We found that gain events were more common than loss events in CNVRs of Meishan pigs, and had larger sizes than losses on average". Is this an observation of other breeds as well? I suggest to tabulate the statistics of CNVRs in other breeds and discuss why this observation makes sense from evolutionary prospective.

Response: Considering the limitation of sequencing depth in detecting CNV, we removed the CNV analyses and this concern is no longer involved in the revision.

I could not find supplementary figures.

Response: This question is no longer involved in the new manuscript.

Lines 276-284: Fst and deltaAF are highly correlated metrics. I suggest removing this paragraph from the main text.

Response: Thank you for your suggestion, we have modified this paragraph on page 9, Lines 10-30 in the new submission.

Line 295: "To distinguish true genes related to high reproduction from genes with homozygosity caused by drift". This is a very strong argument to relate a panel of genes with reproduction performance of Meishan breed without providing significant evidence. The panel of detected genes must be referred as "candidate genes" or "genes putatively under selection".

Response: Thank you for your suggestion, we have applied new strategy to detect selective sweep of meishan population, and this question is no longer involved in the new manuscript.

Line 297: "recovered" or co-localized ?

Response: Thank you for your suggestion, Sorry for our inaccuracy statement, the "co-localized" was better than "recovered" in our manuscript.

Line 340: Is this missense mutation evolutionary intolerant? What is the SIFT value. What is the allele frequency ?

Response: Thank you for your comments. We think that if this missense mutation exists in population, it will be tolerant variation in the evolution. SIFT value was used to predict amino acid changes that affect protein function. The details can be find in PMCID: PMC168916. Allele frequency represents the relative frequency of a SNP or indel allele in the Meishan population.

Line 380: top [candidate] genes.

Response: Thank you for your suggestion, the “candidate genes” is actually better than “top genes”.

Lines 384-387: If the statement "Our finding of greater genetic distance in Meishan pigs as compared to the other Asian local pig breeds and wild boars is consistent with there being a domestication bottleneck caused by human-driven artificial selection and domestication around 4000 ~ 5000 years ago." stands true then what is the explanation for the observed dropping Ne for breeds like Min, Diannan who were bottlenecked at 8000 y.ago or souch China-origin pig at 1000 y.ago? Shouldn't the human population expansion and subsequent domestication affect all pig breeds more or less in the same fashion? What is the unique climate during the Liangzhu civilization that deeply lasted Mishan domestication?

Response: This is a very insightful suggestion. Sorry for our inaccuracy description for the result of the MSMC in our previous manuscript. We speculated that human-driven artificial selection may result in a "bottleneck" in the evolution of different domesticated breeds. Meanwhile, a later bottleneck of Meishan breed was more likely associated with unique domestication process and specific Liangzhu civilization. We reorganized the language to offer the more detailed description in our new manuscript.

Line 449: what was the strategy for samples collection? Are they collected from a single or multiple farms? From where they are taken and was that random?

Response: Thank you for your questions, the strategy for samples collection was that Meishan pig were randomly collected from multiple farms in Kunshan city of Jiangsu province, and Durocs randomly collected from multiple farms in Yancheng city of Jiangsu province.

Fst or FST. Keep consistent.

Response: Thank you for your suggestion, we had unified the "Fst" in our new manuscript.

Reviewer #2: Review report

Domestication bottleneck and genetic divergence ….

The authors investigate Asian and global pig demography, population structure as well as selective sweeps. The paper makes available (I think) a good resource for the community and contains some nice parts. Example, demography, population structure etc.

Response: Thanks for your general positive comments on our works.

However, its analyses of selective sweeps is less successful and the evidence may not support the conclusions drawn from it. In general, the authors identify gene sets and then pick small subsets of genes related to reproduction for further analysis. This seems quite arbitrary. I will outline my concerns below.

Response: Thank you very much for your advice. According to your suggestion, we had applied a new strategy and extremely strict standards to detect selective sweep of meishan population. The result is not arbitrary and well represent a genetic and phenotypic characterization of meishan pig in the new manuscript.

English: The paper would benefit from some English editing.

Response: Thank you for your suggestion. We specially invited the Editage company for offering professional English language editing to this study.

Abstract: Need to define abbreviations

Response: Thank you for your suggestion. We had supplemented abbreviations in the abstract of new manuscript

Intro: Need to define abbreviations. What is a CNVR?

Response: Thank you for your suggestion. We had supplemented abbreviations in the introduction of a new manuscript. CNVR is no longer involved in the new manuscript.

L164: redundant, do you mean overlapping?

Response: Thank you for your suggestion. Sorry for our inaccuracy word and we had changed the "redundant" in our new manuscript.

L172: Actually GW has more duplications than MS.

Response: Considering the limitation of sequencing depth in detecting CNV, we removed the CNV analysis and this question is no longer involved in the new manuscript.

L214: hysteresis ?

Response: Thank you for your question. Sorry for our inaccuracy explanation, we had offered the new description for the Fig.2E in our new manuscript at page 8, Lines 6.

L238+: CNV that are discrete to MS.

You find 1212 genes that overlap discrete MS CNVs. You then pick 19 genes because they fit your preference. I don't think this is rigorous enough. You need to at least demonstrate that this "enrichment" is significant from random.

Response: That is a good suggestion for us. It is actually less rigorous to pick the gene to associate with the phenotypic characterization of meishan pig. Therefore, according to your suggestion and the limitation of sequencing depth, we removed the CNV analysis and this question is no longer involved in the new manuscript.

L259: You calculate Fst and define selective sweeps. You don't account for drift. Is 20% rigorous enough to define peaks? It seems too permissive.

Then 851 genes are identified under peaks and you select 18 of them because they have something to do with reproduction. Is this not just cherry picking results?

Choosing the overlap of SNP and Indel peaks is not really useful either, as drift would affect the same regions.

Response: This is a very meaningful suggestion. According to your suggestion, we had change the criterions of Fst peaks from 20% to 1% for identifying the regions of selective signals. The results is updated in the new manuscript.

L309: Again you have 443 genes and pick 6, because they fit your paradigm. What about the other genes? If you pick 443 genes at random, would you not have at least 6 related to reproduction?

Response: Thanks for your insightful comments! The methods to find the reproduction related gene is really less rigorous! Therefore, we had completely changed the analysis strategy for revealing genetic and phenotypic characterization of meishan breeds. The new analysis strategy and results are updated in the new manuscript.

L319: you then use 21 genes (it is not clearly stated how these are chosen) for network analysis. I assume they are from the analyses above. In the end you identify an interesting variant in NCOA1, but how confident can we be in this result?

Response: these 21 genes were really selected from the analyses above. It does lack confident to directly identify an interesting variant in NCOA1. This question had been corrected and no longer involved in the new manuscript.

L379: You state: "We found that the majority of top genes under selection in Meishan have functions related to reproduction". This is simply false! As shown above, the vast majority have unknown and other functions based on your own evidence.

Response: Thank you for your comments. It was a weak conclusion in our previous manuscript. Now this question is no longer involved in the new manuscript per your comments.

Methods:
You should provide a table of basic stats per animal, including coverage and metadata.
Where will data be made public?

Response: Thank you for your suggestion. We had added the basic information of pig to the Supplemental Table 1.

Reviewer #2: Review report

This paper reports the analyses of 60 new genome sequences of both Meishan and Duroc pigs, together with 59 published genomes. Unfortunately, as it is now, the manuscript is unpublishable given the lack of clarity and misuse of the several statistics used. For instance, differentiation sttaistics such as Fst does not make sense if an unbalanced pools of individuals is used. Further, outgroup species and pig genomes are combined in some analyses where there should be only species. To detect selection, population genetics theory clearly delineates among tests that exploit within species / population variability and between species / populations variability, but authors pool all them without a clear pattern. Furthermore, even if 30 Duroc and 30 MS were sequenced, allowing a neat analyses, a pool of breeds is used without knowing exactly what the DU sequences were used for. Some figures are difficult to interpret or do not match with data provided (eg, in Fig 1A, AS columns are narrow while there should be many more than outgroup GW individuals; in EW, to which breed pertains each individual). I shortlist below a few major criticisms.

Response: Thanks for your criticisms. According to your question and suggestion, we made a great change to our previous manuscript. Most of the problematic parts had revised, such as the pools of breeds; the standards to detect selective sweep of meishan population; the criterion for picking meishan traits related genes; and so on. The new analysis strategy and results is updated in the new manuscript.

1- SNP calling is done on the joint population. This has some advantages but also disadvantages, as rare alleles are to be taken by sequencing errors. Given that sample depth is very different between old and new sequences, there is going to be a strong bias in SNP calling since no specific filtering is done per sample. Further using outgroup samples together with Sus exacerbates this problem, and probably underestimates the genetic differences. The NJ tree in Fig 3 suggest similar distances between Sus and outgroup populations, which make no sense, and warthog is actually closer to pig than say S celebensis, while the evolutive distance with warthog is several million year larger. Some criteria do not make sense, such as filtering by HW equilibrium since you are analyzing a multibreed sample.

Response: Thank you for your suggestion. There is actually a strong bias in SNP calling in our previous manuscript. Therefore, we only reserve three pure populations and add the new SNP filtering criterion to ensure the reliability of new results. The analysis strategy and results are updated in the new manuscript.

2- For Fig 1 it does not make sense to include outgroups. Further, for the heatmap, it would be informative to see the individual genomes, not the average breed. How similar were genotypes from the new and old MS / DUroc sequences?

Response: Thank you for your comments. Sorry for our inaccuracy description in Fig 1. We had updated the new results to our new manuscript. In our new manuscripts, both individuals and breeds would compare with each other. As to the question about the new and old MS / DUroc sequences. we found that there is no difference between the new and old sequences as shown in Fig 3.

3- The introduction is unorganized and some sentences are repeated (eg line 84) . The results should start with a summary of what was sequenced, average depth; Fig 3A should be in the first place to have it in context.

Response: Thank you for your suggestion. We made the corresponding change in the new manuscript based on your insightful comments. The introduction was reorganized and changed some statements that are obscure or less than obvious; As to the results, the first part used to summarize the basic information for our sequenced data and average depth; Fig 3A is no longer involved in the new manuscript.

4- Warning: PC is highly sensitive to unequal sampling, probbaly MS is so separated because of larger N. Which are the Duroc?

Response: Thank you for your warning. We had checked the Duroc and found that the unequal sampling actually effected the separated pattern. Therefore, we applied new strategy in our new manuscript as shown in Fig.2.

5- Sentence in line 147 is unnecessary, evolutive studies much earlier than ref. 16 do show that they are the ancestors or better that they share a common ancestor.

Response: Thanks for this very helpful suggestion. According to your suggestion, we had removed this unnecessary sentence in our new manuscript.

6- The fact that MS have fewer SNPs do not prove that thais breed has lower variability in this design. First, you have to correct by the number of samples. Second, you will find more SNPs when you pool different breeds (say in EW) than when you analyze several together, but all published studies have consistently shown that most Asian breeds have higher variability than say European wild boars or European breeds.

Response: This is a very insightful suggestion. We find that it's not enough to use the number of SNPs to evaluate the variability of breeds, and pooling different breeds together will lead to a bad result. Therefore, according to your suggestion, we had replaced the number of SNPs by genetic distance and linkage disequilibrium (LD) level to evaluate the variability of breeds in our new manuscript.

7- I have found that PSMC is quite sensitive to issues such as depth so all these results should be carefully interpreted.

Response: That is a good suggestion for us. Sorry for our inaccuracy explanation for the result of the MSMC in our previous manuscript. We reorganized the language to offer the careful description in our new manuscript.

8- It is mentioned that 19 genes out of 1212 with CNVs are related to reproduction but is this significant, how many genes are related to reproduction?

Response: Thank you for your suggestion. Considering the limitation of sequencing depth in detecting CNV, we removed the CNV analysis and this question is no longer involved in the new manuscript.

9- The whole section on selective sweeps is unconvincing from the statistical and design point of view. Authors seem unaware of the many different signals that selection can produce. As I mention, Fst analyses between what populations? Both AS and EW are pools of breeds. Why not focusing on DU vs MS at least initially? Do you include the outgroup? tajimas's results are not reported. How do you deal with missing data? Statistics omega should be at least briefly explained, what does it detect? How do you test for significance?

Response: We totally agree with your suggestion! We had removed the AS and EW with pools of breeds, and added 30 Tibetan wild boars as representatives of Asian wild boar population. Meanwhile, we had applied a new strategy and extremely strict standards (Fst peaks from 20% to 1%) to detect selective sweep of meishan population. The result is not arbitrary and well represent genetic and phenotypic characterization of meishan pig in the new manuscript.

No accession is provided for the raw data (or intention of making data available at SRA or equivalent).

Response: Thank you for your suggestion. We had added the basic information of pig to the part

“availability of data and material” in the new manuscript.
