# Supplementary material for: Evidence of evolutionary history and selective sweeps in the genome of Meishan pig reveals its genetic and phenotypic characterization
Source: Gigascience. 2018 May 15;7(5):giy058. doi: 10.1093/gigascience/giy058 (PMC6007440; doi:10.1093/gigascience/giy058)
Supplement: Response_to_Reviewer_Comments_Original_Submission.pdf [file giy058_response_to_reviewer_comments_original_submission.pdf]

Author's Response To Reviewer Comments

Close

March 23, 2018

Re: GIGA-D-18-00018

Hans Zauner
Assistant Editor
GigaScience

Dear Editor and Reviewers,

I am pleased to hear from you and thank you very much for your positive comments, which have improved our manuscript "Evidence of Evolutionary History and Selective Sweeps in the Genome of Meishan Pig Reveals Its Genetic and Phenotypic Characteristics" greatly. We have provided detailed responses to all of your comments point-by-point in the attached pages. For ease of reviewing, all significant changes in the revised manuscript have been highlighted in red.

We also remind you that we have refreshed the contributions of author within the Title Page file, because Xueqin Ran and Jiafu Wang provided language modification in revision.

We hope that our revision has satisfactorily addressed the comments and will now be acceptable for publication in your esteemed journal, GigaScience.

Sincerely,

Jian-Feng Liu, Ph.D.
Department of Animal Genetics and Breeding
China Agricultural University
Email: liujf@cau.edu.cn
Phone No.: 86-10-62731921

Comment:Please follow this example format for the reference:
[xx] Author1 N, Author2 N, AuthorX N. Supporting data for "Title of your manuscript". GigaScience Database 2018. http://dx.doi.orgxxxxxxxxxxxx
(If you don't know the DOI of your GigaDB when submitting the final version, just leave the dummy version above, and we will replace this before publication).

Response: Thank you for your guidance. We have added reference [61] to the last sentence of "Availability of data and material" (page 19) in our revised version, as you have suggested.

Comment: Please also ensure that all data in SRA and other public archives is openly available and that all relevant accession numbers are stated in the manuscript.

Response: Thank you for your guidance. All data for this article in SRA were uploaded into the NCBI database with BioProject ID: PRJNA378496. The release date for the data is April 1, 2018. Thank you for your understanding.

Reviewer #1: GIGA-18-00018

Comment: The authors describe a large collection of pig whole-genome sequences including Meishan, Tibetan wild and Duroc. The paper is much improved over the last version and has changed substantially. The selection signature analysis is much more stringent. Interesting results are highlighted that relate to selection signatures for fertility, black skin and wrinkled skin.

Response: Thank you for your positive comments on our work.

Comments: The manuscripts still requires more English grammar correction and editing.

Response: Thank you very much for your recommendation; the manuscript has been thoroughly

proofread by the authors, and we have made our best effort to make the presentation as professional as possibleThe manuscript has also been edited by a professional editing company. Changes in the revised manuscript are indicated by red highlight.

Specifics:

Comments: Title: should be "Selective Sweeps"?

Response: Thank you for pointing out our mistake. We have corrected ”Selective Sweep” to ”Selective Sweeps” in the title of the revised version.

Comments: P3L3: "... Meishan are strongly..."

Response: Sorry for the incorrect grammar. We have used the verb ”are” in our revised version. Thank you for bringing this to our attention.

Comments: P7L20: genetic commutation ?

Response: Thanks for pointing out our mistake. we already corrected ”genetic commutation” to ”genetic exchange” in the title of revised version.

Comments: P16,L2: which version of Samtools?

Response: Thank you very much for your pertinent remarks. We have added the version of Samtools in the revised version.

Comments: figure quality was not very good, which made an assessment difficult.

Response: Thank you for your suggestion. We have enhanced the figure quality in the revised version.

Comments:This is just a quick follow-up to yesterday's email. I had another look at the supplemental tables, and I noticed that in table S3 some of the genomic regions are indicated as "######". Is this missing data, or can these sequences not be located in the reference genome? Please clarify in the manuscript.

Response: Thanks for your checking. This is no missing data and it is the problem of display in long string. We can view it by extending the table width.

Comments:I recommend that you include a legend / a table of content for your supplemental material at the end of the manuscript, that is, a short description of each supplemental table.

Response: Thanks for your guidance. We have already added the the legend of table to a supplemental material at the end of the manuscript.

Comments: Two other minor points: In the methods section, please mention the software version for all tools used. On page 17 you mention "R packages" used for visualization - please specify the names of these packages.

Response: Thanks for your suggestion. We have already added all the software version number and the R packages for visualization.

Close
